# Toward Standardized Photocatalytic Oxygen Evolution Rates Using RuO_2_@TiO_2_ as a Benchmark

**DOI:** 10.1016/j.matt.2020.07.021

**Published:** 2020-08-05

**Authors:** Hugo A. Vignolo-González, Sourav Laha, Alberto Jiménez-Solano, Takayoshi Oshima, Viola Duppel, Peter Schützendübe, Bettina V. Lotsch

**Affiliations:** 1 Max Planck Institute for Solid State Research, Heisenbergstraße 1, 70569 Stuttgart, Germany; 2 Department of Chemistry, University of Munich (LMU), Butenandtstraße 5–13, 81377 München, Germany; 3 Max Planck Institute for Intelligent Systems, Heisenbergstraße 3, 70569 Stuttgart, Germany; 4 Cluster of Excellence e-conversion, Lichtenbergstrasse 4a, 85748 Garching, Germany

**Keywords:** oxygen evolution reaction, photocatalysis, benchmark, best practice, ^18^O isotope labeling, relative photonic efficiency, quantum yield

## Abstract

Quantitative comparison of photocatalytic performances across different photocatalysis setups is technically challenging. Here, we combine the concepts of relative and optimal photonic efficiencies to normalize activities with an internal benchmark material, RuO_2_ photodeposited on a P25-TiO_2_ photocatalyst, which was optimized for reproducibility of the oxygen evolution reaction (OER). Additionally, a general set of good practices was identified to ensure reliable quantification of photocatalytic OER, including photoreactor design, photocatalyst dispersion, and control of parasitic reactions caused by the sacrificial electron acceptor. Moreover, a method combining optical modeling and measurements was proposed to quantify the benchmark absorbed and scattered light (7.6% and 81.2%, respectively, of *λ* = 300–500 nm incident photons), rather than just incident light (≈AM 1.5G), to estimate its internal quantum efficiency (16%). We advocate the adoption of the instrumental and theoretical framework provided here to facilitate material standardization and comparison in the field of artificial photosynthesis.

## Highlights

• A highly reproducible photocatalyst, RuO_2_/TiO_2_, has been developed as a benchmark system for the oxygen evolution reaction
• A concept of relative photonic efficiency using this benchmark is introduced
• A method to estimate internal quantum efficiency is proposed
• Best practices to ensure the reliable quantification of photocatalytic O_2_ evolution data

## Progress and Potential

The continuous increase of atmospheric CO_2_ levels at alarming rates has put photocatalytic water splitting in the spotlight as a source of “green” energy . However, quantitative comparison of photocatalytic activities across different labs remains challenging, since it involves multiple factors including light scattering and reactor design. Here, we propose two methods to compare photocatalytic performance for the oxygen evolution reaction (OER) across different setups, and put forward a set of good practices to ensure its quantification: first, a benchmark OER photocatalyst (RuO_2_/TiO_2_) for the reliable determination of relative photonic efficiencies, and second, a procedure for estimating internal quantum efficiency by means of optical modeling and light-scattering measurements. These methods will open up new avenues for reliable comparison of photocatalytic performance across different laboratories and give a better overview and insight to the field.

## Graphical Abstract

**Figure:**
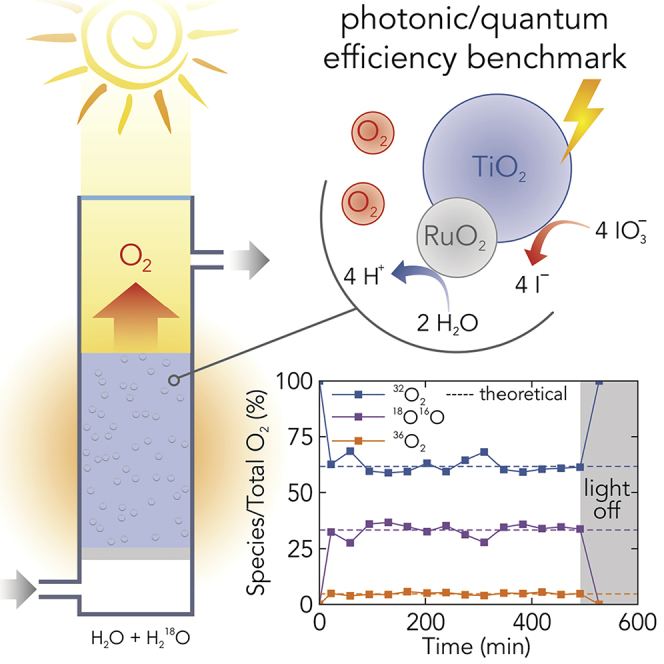

## Teaser

We present an approach to compare intrinsic material efficiencies in the field of artificial photosynthesis by means of highly reproducible photodeposited RuO_2_ on commercial TiO_2_ (P25) as a benchmark system. This is a challenging undertaking for which we present conceptual discussion and offer practical solutions. Here, we put forward two complementary methods (relative photonic efficiency and internal quantum efficiency) to achieve standardization of photocatalytic oxygen evolution reaction rates measured in different experimental setups and present a set of best experimental practices for reliable quantification.

## Introduction

Global anthropogenic CO_2_ emission rates grow at alarming rates. Along with increasing the share of renewables on energy portfolios worldwide, it is projected that renewable-to-chemical energy conversion is essential to control the global temperature rise.^1^, ^2^, ^3^ In this context, artificial photosynthesis has gained attention in the last decades as it tackles larger-scale solar energy storage by producing chemical fuels such as hydrogen from abundant water and sunlight.^4^, ^5^, ^6^ Nevertheless, the pinnacle of artificial photosynthesis, namely photocatalytic overall water splitting (POWS), not only drives an overall thermodynamically uphill chemical reaction using sunlight but also involves the kinetically highly challenging water oxidation reaction. Due to the latter, POWS has been blended with different branches of material research in order to achieve technically feasible applications. Since the first experiments with TiO_2_ in 1972 by Honda and Fujishima that showed OWS for the first time, a large number of other semiconductor materials have been tested for POWS on a lab scale, reaching several milestones in solar-to-hydrogen efficiency (STH). Although the most efficient materials are still relatively far from the commercial target of 2–4 US dollars per kilogram of H_2_, the prospects of POWS using particle suspensions still look promising considering their low cost and STH efficiencies that are nowadays closer to the technical target of 5%–10%.^5^^,^^7^, ^8^, ^9^, ^10^, ^11^, ^12^

Despite the evident progress in this field, a gap persists when comparing photocatalytic STH and other indicators of different materials synthesized and characterized under different groups' standards. An International Union of Pure & Applied Chemistry (IUPAC) report recommends to use so called “internal” quantum yield (Φ), which is defined as the ratio between the number of products formed or reactant consumed and the number of photons absorbed at a certain wavelength (for other definitions of indicators, see the calculations in Experimental Procedures).^13^^,^^14^ However, in heterogeneous suspension systems, it is very challenging to quantify the number of absorbed photons due to light scattering and/or reflection. As a compromise, photonic efficiencies (ξ_e_) or apparent quantum yields (AQY), which consider total incident photons instead of absorbed photons in the yield ratio, are conventionally used. Hence, the key prefix “internal” or “apparent” (or “external”) are used broadly to distinguish between absorbed or incident photons in the denominator. Strictly, the term “yield” should be restricted to the cases where the wavelength of such photons is narrowly bounded (i.e., in the limit of a single, specific wavelength), while “efficiency” refers to polychromatic light. Notwithstanding, photonic efficiency and external quantum efficiency (EQE) have been commonly used as equivalent external indicators to AQY when specifying a wavelength range, and likewise, internal quantum efficiency (IQE) has been commonly used interchangeably with internal quantum yield as an internal indicator.^13^, ^14^, ^15^, ^16^, ^17^ Regardless of the consensus on terminology used to describe photocatalytic activity, along with the problem of light scattering, photocatalytic activity is dependent on many other factors, e.g., light intensity, extinction coefficient, reactants, and reactor design, most of which are either difficult to determine or to standardize.^13^^,^^15^^,^^18^, ^19^, ^20^ These facts blur the current state of the art in the field. As a response to this challenge, several efforts have been made to create a platform for comparing photocatalytic activities under standardized conditions.^15^^,^^17^ For example, a recent work proposed that materials should be compared only by photonic efficiencies at plateaus of photocatalytic production (or consumption) rate versus catalyst loading, which is defined as optimal photocatalytic rate (*r*_opt_), then divided by the total incident photons to calculate the optimal photonic efficiency (ξ_e,opt_).^13^^,^^15^ However, this is a simplistic approach to describe performance as compared with the more reliable internal indicators such as Φ. The Φ of a material is a more complex function of local light intensity at each wavelength, whose integration along the reactor suspension and light spectra gives the total photocatalytic rate observed.^21^^,^^22^ However, assuming such averaging when comparing ξ_e_ at the optimal regime at least gives a glimpse at which material is more active than another overall, including the influence of a specific reaction cell in a regime where light cannot be further extinguished. It must be noted that this optimal ξ_e_ approach does not fully resolve the aforementioned light absorption quantification intricacies, and while technically challenging, internal quantum yield, or alternatively IQE, should be the preferred indicators for material comparison among different groups because such indicators suffer from less influence of the setup on their estimation.^13^^,^^15^^,^^20^^,^^23^

**Figure 1:**
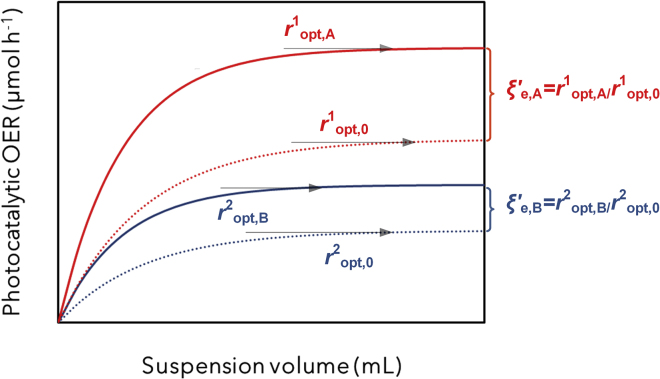
Photocatalytic Activity Versus Photocatalytic Suspension Volume Representation Definition of optimal oxygen evolution reaction (OER) photocatalytic rate (*r*_opt_), and relative photonic efficiencies (ξ′_e_) using a unique illumination standard for materials A, B and benchmark (zero). Adapted from Qureshi and Takanabe.^13^

Alternatively, other novel hardware-based approaches can be also found in the literature to screen Φ directly, such as the integrating sphere embedded reactor^17^^,^^23^ and the black-body reactor.^22^^,^^24^^,^^25^ When compared with the optimal photonic efficiency approach, hardware-based approaches are useful and even more powerful complementary techniques to quantify Φ functions directly if the locally absorbed photon flux profile along the photoreactor cell is accessible. Nevertheless, typical applications of the black-body reactor design suffer from unaccountable light distributions,^24^^,^^25^ which is key to reporting a meaningful Φ function,^21^^,^^25^^,^^26^ whereas the integrating sphere design restrictions in terms of photoreactor design make it less amenable to the comprehensive standardization of photocatalytic rates.^17^^,^^23^ In this context, if Φ (or IQE) quantification is technically not possible, a possible methodology to reflect experimental discrepancies in different groups without imposing a particular photoreactor design is to include an internal standard material in the optimal photonic efficiency approach, assuming that its intrinsic Φ is measurable and universally consistent. To introduce this idea, let us imagine a generic newly developed photocatalyst within a research group 1, namely Material A, which is measured relative to an internal standard (Material “zero”) in a specific setup 1 (*r*^1^_opt,A_ versus *r*^1^_opt,0_) to produce a reaction of interest (i.e., oxygen evolution reaction [OER]) at a reproducible incident light condition *I*_0_ (i.e., AM 1.5G); and a similar study on Material B in a certain research group 2 (*r*^2^_opt,B_ versus *r*^2^_opt,0_), as depicted in Figure 1. If optimal photocatalytic OER rates of both Materials A and B are normalized as *r*′_opt,A_ = *r*^1^_opt,A_/*r*^1^_opt,0_ and *r*′_opt,B_ = *r*^2^_opt,B_/*r*^2^_opt,0_—or, equivalently, ξ′_e,A_ = ξ_e_^1^_opt,A_/ξ_e_^1^_opt,0_ and ξ′_*e*,B_ = ξ_e_^2^_opt,B_/ξ_e_^2^_opt,0_ in the case of Materials A or B using a light standard different than *I*_0_ (from now on defined generically as relative photonic efficiency, ξ′_e_)—and as long as the internal standard has the same Φ property in both cases, the normalized rates *r*′_opt_ show semiquantitatively whether A or B has the higher apparent activity relative to the other material. For this comparison, there would be no other particular constraint on reaction media or reactor design for groups A or B, but general good experimental practices,^13^^,^^15^^,^^27^ and that catalyst A, catalyst B, and the standard 0 are at the optimal point of their respective setups.

In 2003 and subsequently in 2011, similar photocatalysis standardization attempts had been developed in the field of photocatalytic degradation under ISO Standards, whereby acetaldehyde photodegradation on P25 Degussa (Evonik), a commercial form of TiO_2_ (20-nm nanoparticles, 80% anatase, 20% rutile), was used as a standard system.^15^^,^^28^^,^^29^ The foundations of these studies were laid by a comprehensive article by Serpone et al. in 1997 who developed the concept of relative photonic efficiencies (ξ′_e_) providing actual quantitative trends of P25 light absorption and scattering photocatalytic rates, and set the standard terminology for the field.^17^ To the best of our knowledge, however, such methodologies have never been adapted to actual photosynthetic systems, presumably because of the fact that there is an inherent difference between photocatalytic degradation and energy conversion. Unlike photocatalytic degradation, artificial photosynthesis requires an activation step (in other words co-catalyst deposition) that is ideally simple, reproducible (optically and chemically), and robust, i.e., that maintains the nature of its surface chemistry whatever synthetic methodology is followed, which excludes doping or thermal sintering approaches. These restrictions most likely have hindered the concept of a benchmark photocatalyst in the field of artificial photosynthesis.

**Figure 2:**
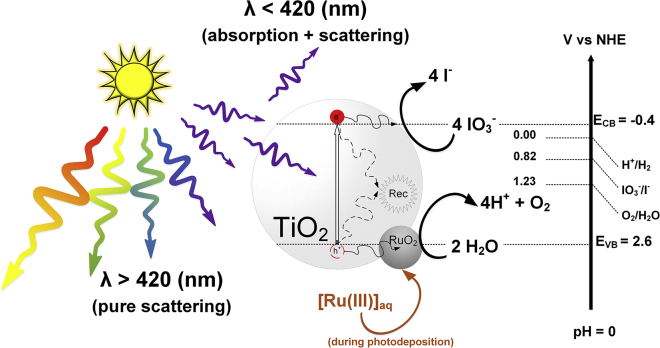
Band Diagram (Not to Scale), Light Absorption, and Reaction Representation of P25/RuO_2_ Benchmark for Photocatalytic OER

Here, we propose RuO_2_ deposited on P25 as a benchmark photocatalyst for OER, which involves a kinetically challenging 4-electron transfer process, as an OER benchmark sufficiently active to estimate the required *r*_opt,0_ reliably, to calculate relative photonic efficiencies as described in Figure 1. P25 as a commercial form of TiO_2_ has been widely used as a photocatalyst including in different direct solar energy conversion studies.^30^, ^31^, ^32^, ^33^, ^34^, ^35^, ^36^, ^37^ P25 eliminates typical reproducibility problems that come with material synthesis because of its standardized fabrication. We identify optimal conditions from literature to deposit RuO_2_ on colloidally stabilized P25 in a highly reproducible manner and thoroughly characterize the benchmark photocatalyst and its OER rates. In addition to the quantification of OER rates of reproducible optimal samples using the optimal photonic efficiency concept, isotopic labeling was performed under *operando* conditions to unequivocally associate such rates with water oxidation, as schemed in Figure 2. This result underpins our suggested best practices, which materialize in a set of recommendations that solve typical reproducibility weaknesses in the field. In addition, light absorption assessment is a feature in photocatalysis that is not frequently included in photocatalyst screening. Here, we present a method to estimate IQE by means of scattered light measurements and optical modeling, which estimated the absorbed and scattered light in the system as depicted in Figure 2. This IQE modeling is a measurement of efficiency that is more reactor independent and is measured under *operando* conditions for this benchmark, which opens a second door to a more powerful way of comparing materials among different groups in the field of artificial photosynthesis.

## Results and Discussion

### Co-catalyst Loading, Dispersion Environment, and Characterization

A catalyst/co-catalyst system that is maximally reproducible, robust, and error-tolerant regarding its synthesis while showing good OER rates is instrumental in its use as a benchmark system for determining relative photonic efficiencies. With this set of requirements in mind, two different deposition methods in liquid suspension were screened using only P25 and a Ru(III) precursor as the light harvester and co-catalyst tandem, respectively: photodeposition (PD) and low-temperature hydrothermal (HT) decoration.^12^^,^^38^, ^39^, ^40^, ^41^ Detailed deposition procedures can be found in Experimental Procedures. Having identified the optimal dispersion condition, the loading amount of Ru was optimized for each method separately (Figure S2). The optimal nominal Ru(III)/P25 weight contents found are 0.15%, 0.30%, and 0.50% for photodeposition (PD), hydrothermal-microwave (HT-MW), and hydrothermal-heating-block (HT-HB) methods, respectively (nominal contents are calculated from initial Ru(III) mass in solution). Real Ru/P25 weight contents are measured with inductively coupled plasma-optical emission spectrophotometry (ICP-OES) analysis (Table S1). According to literature and our UV-visible (UV-vis) experiments (Figure S3), excessive loading of ruthenium (Ru) species leads to a decrease in overall activity due to the black color of the product that competes with P25 light absorption while producing no reaction.^37^^,^^39^ Having identified optimal Ru levels during deposition, we scaled the *ex situ* production of two samples that showed the highest activity at screening conditions, which allows decoupling of Ru deposition from photocatalytic OER rate measurements. These samples were deposited *ex situ* as follows: 0.15% nominal Ru loading using PD in the absence of dispersing agent (Ru0.15/P25-PD) and 0.3% nominal Ru% using the HT-MW method (Ru0.3/P25-HT-MW); for scale-up details of the methods, see Experimental Procedures.

**Figure 3:**
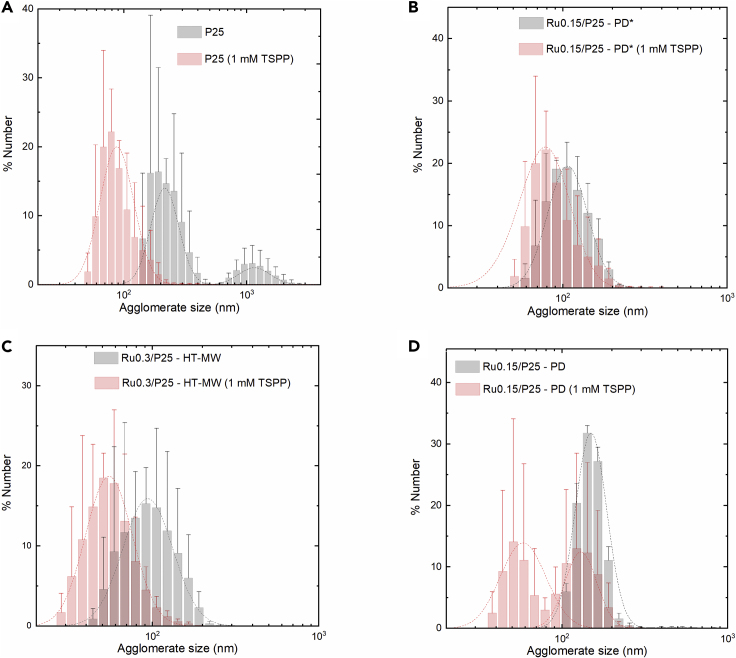
Dynamic Light Scattering % Number Distribution of Agglomerate Size at 0.5 mg mL^−1^ Three candidate P25/RuO_2_*ex situ* deposited samples and P25 suspensions were freshly prepared and sonicated under the exact photocatalytic conditions (10 mM KIO_3_), at 0 and 1 mM TSPP (gray and pink, respectively) to obtain percent number distribution of agglomerate size. Suspension of P25 also contained Ru(III) precursor (0.02 mM RuCl_3_∙*x*H_2_O [0.15% Ru]). Dashed lines are log-normal fittings, and error bars correspond to ± SEM of duplicate datasets (10 redundant DLS measurements each). The *ex situ* deposited samples were previously prepared and collected using the following methods: (A) P25 blank, (B) 0.15% nominal Ru using PD with 1 mM TSPP (PD∗ method, sample Ru0.15/P25-PD∗), (C) 0.3% nominal Ru% using the HT-MW method (Ru0.3/P25-HT-MW), and (D) 0.15% nominal Ru loading using PD in the absence of TSPP (Ru0.15/P25-PD).

A problem usually overlooked in the catalyst deposition on a semiconductor and that we have found much more relevant is the aggregation of semiconductor nanoparticles. The lack of homogeneity in deposition environment should alter the size and/or dispersion of catalyst, and we will show below that achieving a well-dispersed state for the photodeposited Ru species is key for using this system as a benchmark. In the particular case of P25, poor stability in suspension has been evidenced in pure aqueous media at neutral conditions (zeta-potential close to zero).^42^^,^^43^ Accordingly, while depositing catalyst in pure water containing a catalyst precursor, sedimentation of P25 is significant, particularly when the suspension density is close to 1 mg mL^−1^. However, this is a non-trivial problem to tackle, since organic dispersants are undesired due to their hole-scavenging nature. From the inorganic dispersants in literature, tetrasodium pyrophosphate (TSPP, Na_4_P_2_O_7_·10H_2_O) was chosen among other inorganic dispersing agents because of its proven drastic effect in colloidally stabilizing TiO_2_ nanoparticles at relatively low concentrations.^44^ This is crucial, as the agglomerate size and homogeneity affect the dispersion of decorated co-catalyst and the optics of light absorption. In fact, the addition of TSPP visually increases sedimentation time significantly if suspension density is kept around 0.5 mg mL^−1^ of P25 in water.^44^ In addition to this, we also performed dynamic light-scattering (DLS) experiments as a quantitative technique to obtain the %number distribution of different samples in suspension. It can be seen in Figure 3A that the micron-size agglomerates of P25 (alternatively P25 diameter distribution in percent is 1,351 nm [82%], 264 nm [12%], 5,520 nm [6%]) disappeared after the incorporation of 1 mM TSPP, which according to the original study is a result of electrostatic stabilization due to physisorption of highly charged P_2_O_7_^4−^ ions on the P25 surface. On the other hand, excessive addition of P_2_O_7_^4−^ ions to photocatalytic media is not desired either, because it can block active sites by adsorption.^45^^,^^46^ Not surprisingly, the effect of TSPP was found to be beneficial for photocatalytic activity around this optimum concentration of 1 mM but not beyond (Figure S4). We therefore consider the addition of 1 mM TSPP as dispersing agent during photodeposition as a variation of the PD method, from now on defined simply as PD∗. Accordingly, a third *ex situ* sample was produced at 0.15% nominal Ru using PD with 1 mM TSPP as dispersing agent (Ru0.15/P25-PD∗).

The addition of TSPP had a positive impact on dispersion not only during co-catalyst deposition (Figures 3A and S4) but also after the deposition. The qualitative effect of dispersing agent on agglomeration of the *ex situ* sample suspensions was measured by DLS and is presented in Figures 3B–3D for the samples PD∗, HT-MW, and PD, respectively (gray distribution is no TSPP, pink distribution is 1 mM TSPP). These results show that after *ex situ* deposition, the addition of TSPP in all three samples is still beneficial in diminishing agglomerate size during the photocatalytic OER activity measurements.

**Figure 4:**
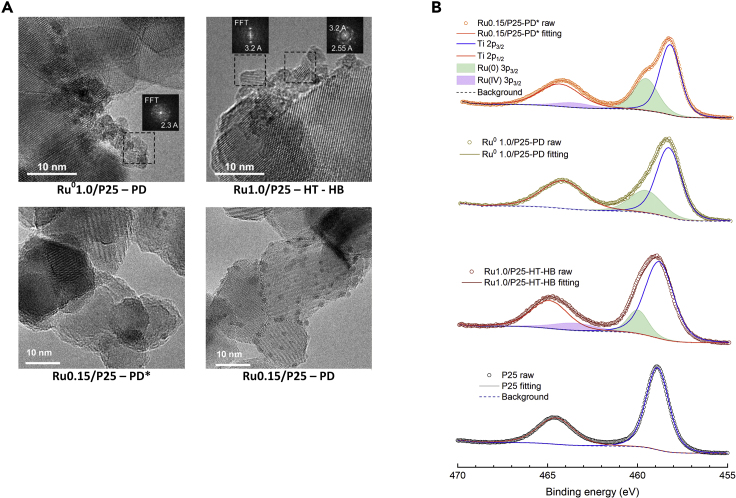
Heterojunction Characterization of P25/RuO_2_*Ex Situ* Deposited Samples (A) TEM images showing the d-spacings obtained by fast Fourier transformation of the regions containing metallic Ru^0^ (2.3 Ǻ) and RuO_2_ (3.2 Ǻ) nanoparticles on P25. Reference samples are 1% PD Ru in methanol medium (Ru^0^1.0/P25-PD), and HT deposited RuO_2_ on P25 (Ru1.0/P25-HT-HB). TEM-EDX data of both are presented in Figure S6. Ru0.15/P25-PD∗ and Ru0.15/P25-PD are the Ru% optimal samples PD *ex situ* with and without TSPP, respectively (scale bars, 10 nm). (B) XPS spectra of Ru^0^1.0/P25-PD, Ru1.0/P25-HT-HB, Ru0.15/P25-PD∗, and P25 blank.

To thoroughly characterize the benchmark material candidate, we used a comprehensive set of characterization techniques including powder X-ray diffraction (PXRD), transmission electron microscopy (TEM) coupled with energy-dispersive X-ray spectroscopy (TEM-EDX) and fast Fourier transformation (TEM-FFT), and X-ray photoelectron spectroscopy (XPS). As mentioned earlier, the PXRD pattern (Figure S7) of P25 confirms that it is a mixture of both anatase and rutile phases of TiO_2_. On the other hand, no apparent change was observed in the PXRD patterns of PD∗ and HT-HB, which indicates low overall amounts of deposited Ru species on P25. TEM images of all the materials along with the TEM-FFTs of PD and HT-HB are shown in Figure 4A, and the corresponding TEM-EDX data are presented in Figure S6. The TEM images underline the well-dispersed nature of the deposited Ru species. Especially the addition of TSPP during photodeposition made larger Ru particles scarcely visible on TEM images. As both samples PD and PD∗ have a similar real Ru content (ICP-OES, Table S1) after deposition, it can be hypothesized that the sample using TSPP during photodeposition had a larger active surface of Ru species than the one without TSPP. The larger agglomerate size of P25 without TSPP restricted the Ru species to form only at the outer surface of those agglomerates, forming larger-sized clusters observable in the TEM as nanoparticles (Figure S8). In contrast, at smaller P25 agglomerate sizes, the Ru species are formed on a larger surface area exhibiting smaller characteristic particle sizes (subnanometer). The d-spacings determined from the TEM-FFTs indicate deposition of metallic Ru and rutile RuO_2_ nanoparticles using PD and HT-HB, respectively (samples Ru^0^1.0/P25-PD and Ru1.0/P25-HT-HB).^47^^,^^48^ In agreement with the above observations, XPS reveals the presence of only metallic Ru (Ru(0)) in PD, but the presence of both Ru(0) and Ru(IV) are observed in samples HT-HB and Ru 0.15/P25-PD∗ (Figure 4B; for detailed analysis see Supplemental Information). No trace of P, Cl, K, or Na was observed by the XPS peak analysis or ICP-OES measurements. Competing Ru(III) reduction to Ru(0) (at ∼0.5 eV versus normal hydrogen electrode [pH 0]) is possible through the photoinduced formation of electron-hole pairs and the relatively small concentration of sacrificial electron acceptor (SEA) during the photodeposition process.^49^

### Photocatalytic OER Detection Platform

**Figure 5:**
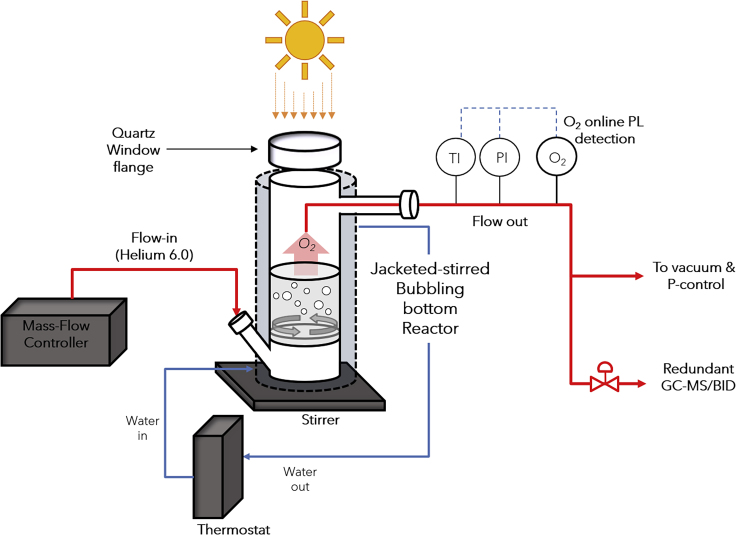
Schematic Experimental Setup for Direct On-Line Quantification of Photocatalytic OER Rates Red lines are inert gas pathways. A controlled helium flow of 10–20 NmL min^−1^ bubbles through the liquid hold-up of the reactor through a porous frit, which is stirred and irradiated with a Xe lamp using an AM 1.5G spectra filter and an incident light intensity of 100 mW cm^−2^.

To allow for the precise quantification of OER photocatalytic rates on *ex situ* deposited RuO_2_ on P25 samples, we developed a continuous-flow high-purity glass reactor^50^ suitable for OER kinetic studies, optimized with a specific focus at overcoming O_2_ mass transfer limitations, air leakages, and dead volumes (Figures 5 and S16). We also explored reactor-engineering concepts to optimize and combine trace detection and on-line analysis, which is flexible and sensitive enough to sample a wide range of reaction rates and reactor geometries; from an optics perspective, it is also simple enough to reproduce and model photon fate. The minimum O_2_ detection limit of the three detection devices at the reactor outlet is around 1 ppmv, which is measured on top of a consistent background amount of around 5 ppmv (for details see Experimental Procedures). At typical flow rates of 10–20 NmL min^−1^, the minimum detectable activity can be as low as 0.02 μmol_O2_ h^−1^, with an instrumental time resolution of 3 s. To the best of our knowledge, a comparably sensitive and versatile design has not yet been reported for photocatalysis, and resembles just vaguely other prototypes in the literature that also explored some forms of bubble-induced on-line photocatalytic OER.^51^^,^^52^

It is worth noting that the high sensitivity of our setup allowed us to identify several OER quantification abnormalities that may disguise the actual activity of P25. For instance, sodium persulfate and silver nitrate are widely used sacrificial electron acceptors; however, we observed O_2_ evolution even without light absorber under illumination (Figure S1). Compared with the measured OER rates in the case with light absorber (bare P25), the fraction of that OER rate evolved in the absence of light absorber was 72% and 0% for persulfate and KIO_3_, respectively. Because AgNO_3_ produces significant optical changes through Ag^0^ deposition during its photoreduction, it was not considered for Ru deposition in this study, yet we quantified its OER background in the absence of P25 and obtained an amount similar to the one of persulfate, using an alternative detection of dissolved O_2_ in liquid (for details of O_2_ detection see Experimental Procedures). The parasitic O_2_ amounts detected in the presence of persulfate and AgNO_3_ are far beyond our instrumental error; therefore, it likely results from spontaneous SEA decomposition.^53^ After trying different combinations of blanks and light conditions, we have identified that moderate KIO_3_ concentrations (<20 mM) and AM 1.5G (*I*_0_) have no significant background except in the presence of bare P25. Additionally, KIO_3_ is not only an SEA but also a potential redox/shuttle for future photocatalytic OWS studies.^54^ This is why we chose this diluted aqueous KIO_3_ solution as a standard reaction medium.

### Optimal Photonic Efficiencies and Reproducibility of Optimal Samples

**Figure 6:**
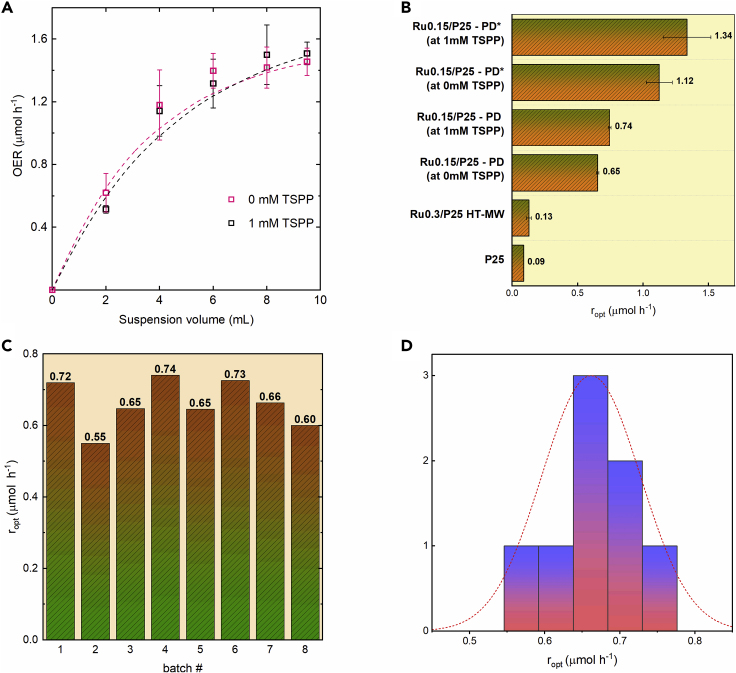
Comparison of Photocatalytic OER Methods and Reproducibility (A) Characteristic curve of optimal sample Ru0.15/P25-PD∗ (produced *ex situ* using 1 mM TSPP, details in Experimental Procedures), at 0.5 mg mL^−1^ and AM 1.5 conditions, at 10 mM KIO_3_ using two levels of dispersant (dashed lines are fitted exponential apparent extinction curves, and data points are presented as mean ± SEM of duplicated measurements). (B) Comparison of overall activity using other deposition methods using the *r*_opt_ definition (error bars represent mean ± SEM of duplicated measurements). (C) Batch-to-batch variation of photocatalytic activity of sample Ru0.15/P25-PD produced *ex situ* (details in Experimental Procedures), then tested at 0.5 mg mL^−1^ and AM 1.5 conditions, at 10 mM KIO_3_ and no dispersant. (D) Histogram representation of batch-to-batch variation in photocatalytic activity of sample Ru0.15/P25-PD (mean and SD values of fitted normal distribution in red dashed line are 0.65 and 0.05 μmol h^−1^, respectively).

Using the setup described in Figure 5, the full curve of optimal photonic efficiency (equivalent trend to optimal photocatalytic OER rate *r*_opt_) of the sample Ru0.15/P25-PD∗ (*ex situ* photodeposited at 1 mM TSPP) is presented in Figure 6A, and a summary of *r*_opt_ of all *ex situ* samples using the same methodology is shown in Figure 6B. The suspension conditions are 10 mM KIO_3_, using the 0 and 1 mM of TSPP levels that were screened before to show differences on the sample agglomerate size (Figure 3), and a unique *I*_0_ illumination standard (AM 1.5G). The sample Ru0.15/P25-PD∗ was preliminarily screened as the benchmark candidate, which is now justified given it has the highest *r*_opt_ measured at 1 mM TSPP (but not beyond, see Figure S4); from now on this set of suspension conditions is simply referred to as benchmark standard.

From these results, it is evident that the optimal photonic efficiency of Ru species formed on Ru0.15/P25-PD∗ (photodeposition using 1 mM TSPP) and Ru0.15/P25-PD (photodeposition without TSPP) are far larger than those observed when using HT deposition methods, the latter being only 44% better than P25 without co-catalyst. The better results for the PD∗ over the PD method were already explained by agglomerate size measurements (DLS) on P25 with and without dispersing agent (Figure 3A), and further supported by TEM. A higher dispersion of the co-catalyst was seen in TEM images using TSPP (Figure S8). On the other hand, the use of TSPP at 1 mM (but not beyond, see Figure S4) versus 0 mM concentration during OER photocatalytic activity measurements has a minor positive impact only on photonic efficiency, on both Ru0.15/P25-PD and Ru0.15/P25-PD∗ samples, which was also supported by the small differences in agglomerate size of the samples observed by DLS when using dispersing agent (Figures 3B and 3D); thus, this impact was expected to be less significant. This is consistent with our observations that the drastic change of distribution of Ru species formed at drastically different agglomerate sizes (Figure 3A) is what makes the sample Ru0.15/P25-PD∗ almost twice as active as Ru0.15/P25-PD. Therefore, the choice of Ru0.15/P25-PD∗ as the optimal benchmark is justified because of photonic efficiency and sample homogeneity. The use of dispersing agent during photocatalytic OER rate measurements is still positive due to the additional colloidal stabilization and higher photonic efficiency, and is thus justified to be included as part of the benchmark conditions (later justified for IQE estimations also).

More importantly, the PD method proved highly reproducible and robust to slight changes in setup during *ex situ* deposition (such as suspension volume, reactor geometries, and different AM 1.5G solar simulators). Although not optimal compared with PD∗ from an activity or dispersion point of view, the batch-to-batch variation statistics of the sample Ru0.15/P25-PD already shows a narrow normal distribution in Figures 6C and 6D. On the other hand, a batch duplicate using the PD∗ method showed a difference in the *r*_opt_ experiment (at benchmark conditions) of only around 4%, and hence even better reproducibility than the PD method.

### Benchmark Photocatalytic Water Oxidation Performance

**Figure 7:**
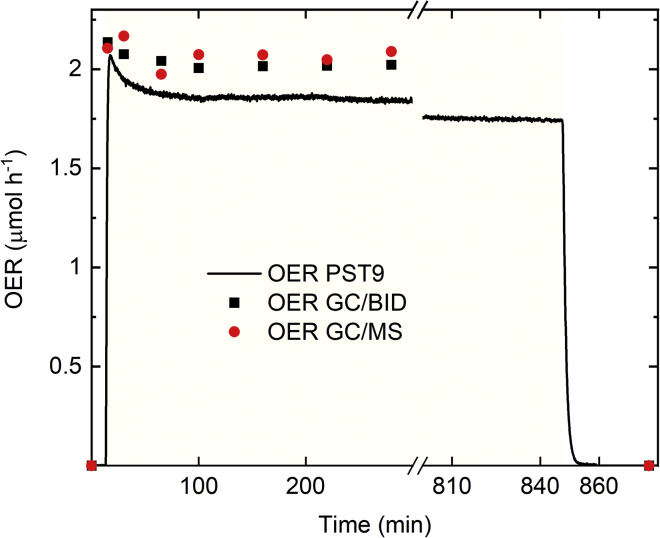
Quantitative Analysis of the Complementary GC-MS-BID System with Longer Time On-Stream AM 1.5G, 1-sun illumination, 5 mg Ru0.15/P25-PD∗, 10 mL H_2_O (10 mM KIO_3_, 1 mM TSPP). After 14 h illumination (yellow graph background indicates light-on time interval), max. TOF_avg_ 49 h^−1^, final TON_avg_ ≈ 600, final TOF_avg_ = 86% of max. TOF_avg_. Max. observed ξ_e_(AM 1.5G) = 0.2%, ξ_e_(AM 1.5G, *λ* < 420 nm) = 3.8%, max. observed IQE (AM 1.5G) = 16%.

**Figure 8:**
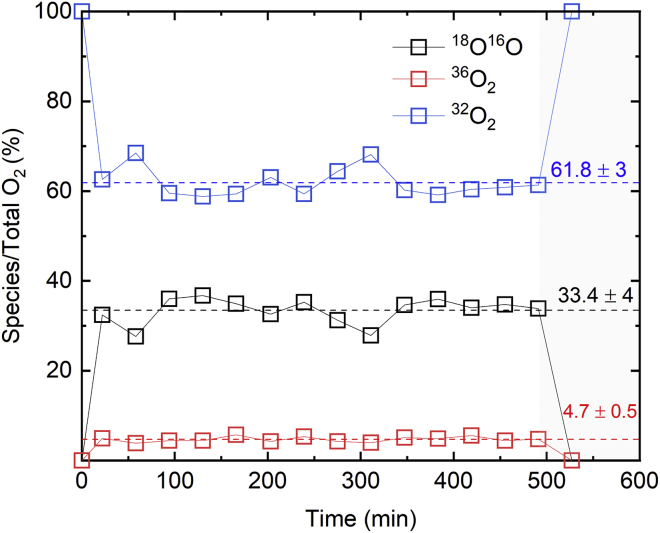
Isotopic Mass Distribution at O_2_ Retention Time of GC For channels *m*/*z* = 32 (^32^O_2_), *m*/*z* = 34 (^16^O^18^O), and *m*/*z* = 36 (^36^O_2_), obtained by integration of molecule counting around O_2_ retention time, after 12 min of sample illumination during 500 min under identical conditions (gray graph background indicates light-off time interval), but 4-mL suspension using a ratio of 1:3 in weight of H_2_^18^O (97%^18^O) to H_2_O (in dashed lines, O population statistic of mean ± SD).

Based on its high reproducibility and external photonic efficiency, Ru0.15/P25-PD∗ was chosen as a suitable OER benchmark. We present its dynamic rate measurements along with other performance indicators at photocatalytic conditions for longer time on-stream (14 h) in Figure 7. The results show no major deactivation in time and also a turnover number (TON_avg_) of over 600 when the light was turned off, which is roughly one order of magnitude higher than the TON of a similar OER model system using a dye photoabsorber and a molecular water oxidation catalyst, near its full deactivation point.^52^ This hints that the RuO_2_/P25 tandem of our proposed benchmark is self-corrosion and agglomeration resistant at this timescale. A drop of 12% activity after the first 20 min is observed, which is usually ignored in the *r*_opt_ calculations because only the final stable values of OER rates are recorded in such cases. This drop might be attributed to I^−^ being backward oxidized.^,^^55^ We also present quantitative analysis using two complementary detection methods for O_2_ by means of gas chromatography (GC). The signal of O_2_ was quantified by highly sensitive barrier discharge ionization detection (BID) and mass spectrometry (MS), whose net response was different by only 8% and 10%, respectively, from the one measured with a photoluminescence sensor that quantifies O_2_ based on the effect of dynamic luminescence quenching by molecular oxygen (PST9); this instrumental error of the three complementary detection devices was included in the statistics of *r*_opt_.^50^ Additionally, using this same platform a quantitative *operando* isotope labeling experiment was included using H_2_^18^O water in a ratio 1:3 with respect to regular Milli-Q water. This experiment is key to unambiguously and quantitatively confirming that nearly all of the oxygen measured indeed comes from water oxidation. Similar attempts were tried in previous photocatalytic water oxidation studies, yet in our experiments a quantitative O_2_ species population analysis in realistic reaction medium is provided.^56^^,^^57^ The population of O_2_ species presented in Figure 8 (^32^O_2_ = 61.8%, ^16^O^18^O = 33.4%, and ^36^O_2_ = 4.7%) is fairly similar to the theoretical one assuming a mean field approximation and no kinetic isotope effect (^32^O_2_ = 60.2%, ^16^O^18^O = 34.7%, and ^36^O_2_ = 5%). Although in terms of relative ratios of ^16^O^18^O and ^36^O_2_ labeled species to ^32^O_2_ this difference corresponds to 20% and 30% error respectively, showing a slight overpopulation of regular ^32^O_2_, this deviation can be corrected considering a kinetic isotope effect (KIE). The isotope effect is predicted by literature placing the rate-limiting step on the direct nucleophilic attack of the H_2_O molecule to the photogenerated hole at surface lattice O sites, in which case both relative ratio errors drop to less than 2% and thus is contained in the instrumental error (Table S3).^58^^,^^59^ This result indeed validates all the blanks and choices made during the development of this methodology, including leakage and reaction control of the SEA.^53^ Note that our RuO_2_/P25 is active for OER not only with KIO_3_, but also other commonly used SEAs such as Na_2_S_2_O_8_ and AgNO_3_, which opens up a platform for other groups to compare photocatalytic performances with an arbitrary SEA.

### Benchmark Quantum Efficiency

**Figure 9:**
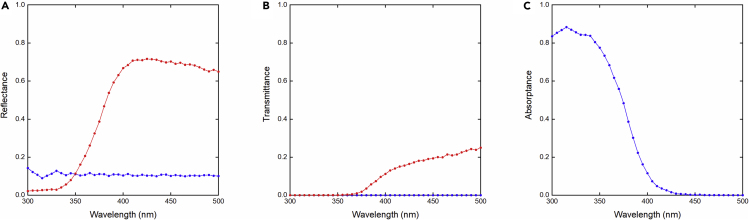
Predicted Photon Fate versus Wavelength in Real Reaction Media (A) Theoretical specular (blue) and diffuse (red) reflectance, (B) ballistic (blue) and diffuse (red) transmittance, and (C) absorptance. Suspension absorptance was used to calculate the rate of photons absorbed by the suspension for IQE calculations.

While the use of relative photonic efficiencies as reporting standard allows for a better cross-laboratory comparability of photocatalytic rates, IQEs have the advantage of being a more objective measure of a material's intrinsic photocatalytic activity, as the effect of scattering/reflection is excluded. As such, the difference between photonic efficiency and IQE signifies limitations of the overall reaction system in terms of light-management and suspension properties, which can then be optimized separately. The IQE (see calculations in Experimental Procedures) of the benchmark sample under standardized conditions was estimated using stochastic optical modeling of the photon fate in the reaction system validated with *operando* measurements of side-scattered light. This is a key estimation that not only tells us about a measure of efficiency of this benchmark that is more independent of the light-management design of the setup but also provides an additional criterion for standardization. Based on this model, the predicted side-scattered photon probability at different wavelengths and depths from the liquid-gas interface (0–32 mm, scheme in Figure S12) is contrasted with experimentally measured light profiles under *operando* conditions, together with incident light *in situ* before the measurement, using a spectrophotometer, which is presented in Figure S13. These results show a good agreement between the theory and experimental values for this section of the reactor, without any type of curve fitting besides position fine adjustment and normalization. The trend obtained for this section is extrapolated to the rest of the geometry and presented as overall probabilities of an incident photon to be reflected, scattered, or absorbed, which is presented in Figure 9.

The fraction of light that is absorbed by the suspension is only around 8.9% (Figure S15). When quantified as absorbed photon flux, the IQE (AM 1.5G) of the system for this reactor geometry is estimated as 16%, versus the estimations for ξ_e_(AM 1.5G) = 0.2% and ξ_e_(AM 1.5G, *λ* < 420 nm) = 3.8%. Notwithstanding, this result should be taken as a spatial and spectral average IQE at this very AM 1.5G spectrum, geometry, and light intensity, since the actual quantum yield Φ of the material is a function of wavelength and local absorbed photon rate. This can be seen specifically from Figure S14 showcasing that most of the photon absorption below 400 nm takes place in the first 2 mm of suspension, and we can expect that the observed IQE is an integration of the contribution of each slice of suspension at different wavelength and light intensities.^21^^,^^26^ Additionally, it can be seen in Figures S15 and 6A that the IQE measured at 10 mL is not a constant, because when compared with the trends of solar spectrum weighted integrated absorptance (SSWIA), defined as

$$\text{SSWIA}=\frac{\int_{300}^{500}A\left(\lambda \right)\text{AM}1.5\text{G}\left(\lambda \right)\text{d}\lambda }{\int_{300}^{500}\text{AM}1.5\text{G}\left(\lambda \right)\text{d}\lambda },$$

where *A*(*λ*) is the absorptance of the suspension predicted for the points of *r*_opt_ measured at less suspension volume, it is clear that the ratio of *r*_opt_ to SSWIA is not preserved, which hints at the expected dependence of IQE on the local light intensity. In practical terms, though, even if what is preserved is the Φ function of the benchmark rather than a constant IQE, this IQE number (16%) may still be roughly extrapolated to other setups where the light absorption profile of a suspension or homogeneous solution can also be quantified (i.e., using an integrating sphere), as a complementary and more meaningful comparison to the proposed ξ′_e_ normalization. The normalization based on relative photonic efficiencies ξ′_e_ proposed here simplifies the comparison of photocatalytic activities obtained for different materials tested in different experimental setups because it captures overall differences in light absorption and scattering for both material and benchmark, as long as the Φ of the benchmark is preserved. On the other hand, the IQE of the benchmark reported here is a trend that is more likely to be preserved quantitatively regardless of slight differences in experimental setups. While Φ is technically challenging to be measured for most heterogeneous suspensions, we still recommend that IQEs should be primarily compared if possible, as IQE is an indicator closer to Φ than ξ_e_ and involves less averaging, and thus is less influenced by the reactor design, which is ultimately desired for a more rigorous standardization among groups.

### Concluding Remarks

We have established a photocatalytic OER benchmark system based on a photodeposition method to produce a material with appreciable and reproducible OER activity, obtained from a suitable commercial reactant that is widely accessible. Using a modified photodeposition protocol complemented by colloidal stabilization under the optimized conditions (loading of 0.15 w/w % of Ru(III) to P25, a suspension density of 0.5 mg of P25 per mL of an aqueous solution containing 10 mM KIO_3_ as SEA and 1 mM TSPP as a dispersing agent), the collected material can be used as a photocatalytic OER benchmark in an activity screening platform by normalizing the photocatalytic OER rates of an arbitrary sample in an arbitrary setup against this benchmark measured under the same experimental conditions. Equally important, we present a set of best practices in the field to make photocatalytic OER quantification in general more reliable, including on-line OER detection, accurate subtraction of leakage backgrounds, elimination of parasitic O_2_ background and minimization of sacrificial use, suspension stability measures, and complementary OER measurements to validate quantification. Importantly, we reveal that typical SEAs such as persulfate and silver nitrate can evolve O_2_ even without light absorber, which advocates for a more careful use of SEAs in general. Finally, we present two novel *operando* techniques to corroborate first that O_2_ was produced solely from water oxidation (^18^O isotope labeling using on-line GC-MS) and that the interplay of light scattering and absorption plays an important role in determining measured photonic and internal quantum efficiencies of the benchmark, as verified by photon fate modeling and side-scattered light measurements. Both techniques play a central role in the determination of the IQE of the benchmark, which is an indicator closer to quantum yield, the gold standard in photocatalysis. Although not much literature is devoted to quantitatively accessing quantum yields in heterogeneous photocatalysis, this is the ultimate performance indicator for the field of artificial photosynthesis because it is reactor independent. We recommend the direct comparison of quantum yield or, alternatively, IQE, to primarily evaluate photocatalyst OER efficiencies, which can be benchmarked with the IQE estimations presented here. Alternatively, for materials whose light absorption cannot be readily quantified, the relative photonic efficiency method presented here is a first step toward standardizing photocatalytic activity results. Therefore, reporting relative photonic efficiencies would greatly simplify the comparison of results reported by different groups. It is our hope that the methodology presented here fosters and facilitates both the creation and use of photocatalytic benchmarks and at the same time opens up new avenues to reliably compare photocatalytic performance indicators across different laboratories by means of quantum yield estimations or, alternatively, IQE.

## Experimental Procedures and Calculations

### Resource Availability

#### Lead Contact

Further information and requests for resources and reagents should be directed to and will be evaluated by the Lead Contact, Bettina V. Lotsch (b.lotsch@fkf.mpg.de).

#### Materials Availability

Generated benchmarks materials presented in this study may be made available and sent on reasonable and well-founded request, but we require prior formal contact and discussion, and we reserve the right to refrain from sending materials to anyone. Requests should be directed to the lead contact (b.lotsch@fkf.mpg.de).

#### Data and Code Availability

The published article includes all experimental data generated and/or analyzed which are necessary to support all observations and conclusions during this study.

### Reactants

Aeroxide P25 (formerly TiO_2_ Degussa P25) nanoparticles (Evonik, Sigma-Aldrich) were pre-treated before and after Ru deposition in a vacuum oven at 60°C for at least 8 h to remove the presence of potential physisorbed organics. Fresh P25 stock was then stored under regular clean conditions. RuCl_3_‧*x*H_2_O (Alfa Aesar, 99.99%) was used as a precursor for both hydrothermal and photodeposition methods. Different amounts of precursor were weighed and stored in an inert atmosphere. Stock solution of Ru precursor was then prepared at 1 mg mL^−1^ and kept no longer than 1 month to ensure stability. TSPP (Na_4_P_2_O_7_·10H_2_O; Supelco) stock was prepared at a concentration of 500 mM. For photocatalytic testing, potassium iodate KIO_3_ (Aldrich, 99.5%) or sodium persulfate Na_2_S_2_O_8_ (Aldrich, 98%) were weighed directly before the experiment together with the sample. Stock solutions were prepared with Milli-Q water.

### Ruthenium Deposition

For hydrothermally RuO_2_ deposited on P25 nanoparticles, 20 mL of Milli-Q water and 20 mg of P25 were stirred and ultrasonicated for 15 min in a microwave vial with magnetic stir bar. Ru stock solution was then added at different amounts and in duplicates, and the vials were closed.^40^^,^^41^ Two different methods were employed. For the microwave method (HT-MW), vials were prepared and placed one by one in a microwave synthesis oven (Biotage Initiator+) at 150°C for 10 h. Using the second method, vial suspension samples and its duplicates were placed all together at the same temperature and time in a home-made heating plate with stirrer and a heating block. For *ex situ* photodeposited samples (PD), two different scaled photoreactor cells were employed to replicate literature methods: the first was 35 mg of P25 in 70 mL of Milli-Q water at 10 mM KIO_3_ at the optimal Ru content; the second was an identical suspension but with the volume scaled to 500 mL.^12^^,^^38^^,^^39^ Solutions were sonicated for 15 min before entering the cell. The purging procedure of the photoreactor cell was identical to that reported in our previous publication, but with the difference of a degassing bubbling line entering the stirred liquid medium to degas O_2_ to the headspace in continuous flow and track the produced O_2_ with a photoluminescence sensor (PST9 flow-through photoluminescence sensor probe + Fibox [Presens]), and some modifications that facilitated powder recovery at the end of the experiment.^50^ Once the headspace purging was finished, the bubbling was started and the O_2_ was monitored until the baseline was reached, after which the light was turned on (Newport, housed Xe lamp simulator). The irradiation condition is a standard ACB quality simulated AM 1.5G, 1-sun condition (Figure S15). The solution was irradiated until the OER rate maximum remained constant for around 30 min, and the suspension was recovered. Typically, the induction time for the Ru precursor to be completely deposited is around 0.5 min mL^−1^ of suspension. For metallic Ru-deposited samples, the same cell as that of our previous publication was used but with 16 mL of Milli-Q water, 4 mL of methanol, 10 mg of P25, and Ru precursor amount. Reaction was stopped when the hydrogen evolution reaction (HER) maximum was obtained and kept for 1 h tracking of H_2_ with the BID detector of the on-line GC analysis. Finally, for all the methods (PD, HT-MW, and HT-HB), the samples were centrifuged in Teflon tubes at 15,000 rpm and then washed/suspended with Milli-Q water and ultrasonicated briefly, repeating this cycle three times. On completion, the centrifuged slurry was dried off overnight in a vacuum oven, mortar ground, and stored in glass vials sorted by batches. P25 controls were produced by using no Ru precursor and following the entire procedure described above.

### Sample Characterization

UV-vis spectroscopy (Cary 5000, Agilent), ICP-OES analysis (Vista Pro Axial, Varian), TEM (Philips CM30 ST, 300kV, LaB_6_ cathode), TEM-FFT, and TEM-EDX (Noran System Seven Si(Li) detector) were performed as previously reported by our group.^50^^,^^60^^,^^61^

DLS measurements were performed using Malvern Zetasizer-Nano equipment, with 13 runs per measurement after full decay of the measured autocorrelation function and after ensuring suitable polydispersity. Each measurement was performed in duplicates using freshly prepared suspension exactly as for photocatalytic activity testing.

PXRD patterns were collected at room temperature on a laboratory powder diffractometer in Debye-Scherrer geometry (Stadi P-Diffraktometer [Stoe]), Cu-Kα1 radiation from a primary monochromator. The samples were sealed in 0.3-mm diameter borosilicate glass capillaries (Hilgenberg glass no. 0140), which were spun during the measurements. Each pattern was measured in a 2*θ* range from 5° to 80° applying a total scan time of 1 h.

For XPS, measurements were carried out in a Thermo VG Thetaprobe system (Thermo Fisher Scientific, USA) employing Al Kα radiation (*hν* = 1,486.68 eV) produced with an electrical power of 100 W. The X-ray spot size on the sample was about 1 cm in diameter. The analyzer aperture circle on the sample was approximately 1 mm diameter. During measurements, the base pressure of the XPS was 1 × 10^−9^ mbar. To compensate for possible peak shifts originating from surface charging, we used Ar ions from a flood gun. The necessary Ar flux inlet was set to a chamber pressure of 3 × 10^−7^ mbar. Survey spectra were recorded with a pass energy of 200 eV, and more detailed spectra were carried out with a pass energy of 50 eV and step width of 0.05 eV. The Ti 2p and Ru 3p peaks were measured with 100 scans to reduce the noise to an acceptable value. The C 1s and the O 1s peaks were measured with 60 scans. All binding energies were calibrated with respect to the C 1s peak position. The measurements were fitted using the fitting routines included in the XPS software Avantage and CasaXPS.

XPS peak analysis was conducted in relation to deconvolution of peaks shown in Figure S5, Table S2, and Figure 4. As the Ru 3p core level XPS spectra overlap with the Ti 2p signal, the spectra of the Ru^0^ 1.0/P25-PD, Ru1.0/P25-HT-HB, and Ru0.15/P25-PD∗ were compared with the blank P25 (Figure 4B). The Ti 2p_1/2_ and Ti 2p_3/2_ peaks of P25 appear at 458.6 eV and 464.6 eV, respectively, which is in agreement with literature reports.^62^ Only a single Ru 3p_3/2_ peak is observed in Ru^0^ 1.0/P25-PD at 459.8 eV, which is likely due to Ru(0). In contrast, the spectra of the PD and HT samples can clearly be deconvoluted to more than two peaks, which are observed due to the presence of Ru in these samples in different oxidation states. In Ru1.0/P25-HT-HB, two Ru 3p_3/2_ peaks appear at 460.0 eV and 463.5 eV, which indicate the presence of both metallic Ru (Ru(0)) and Ru(IV) in the sample.^63^ The spectrum of Ru0.15/P25-PD∗ exhibits two Ru 3p_3/2_ peaks centered around 459.7 eV and 463.7 eV, which support the presence of both Ru(0) and Ru(IV) in the sample. It can be argued that the presence of traces of metallic Ru could indirectly alter the intrinsic activity of the RuO_2_/P25 junction. However, we photocatalytically measured P25 blanks modified with metallic Ru (Figure S1A), finding almost no difference compared with non-modified P25. Furthermore, as the sample Ru0.15/P25-PD and Ru0.15/P25-PD∗ were thoroughly surveyed during TEM imaging, along with the observed RuO_2_ species, a few rare and isolated spots of photocatalytically inactive amorphous metallic Ru were identified on the sample Ru0.15/P25-PD (Figure S6). RuO_2_ is the active species responsible for OER activity in both HT and PD methods. Besides a higher active surface area of the material obtained by the PD method, the PD samples may be more active because of their lack of crystallinity when formed at room temperature and the fact that they are highly dispersed. In fact, after heat treatment (200°C, 2 h in air) of Ru0.15/P25-PD samples, we have found that photocatalytic OER activity drastically drops to the same levels of the HT samples, as this treatment is associated with further RuO_2_ crystallization in some literature concerning this junction.^41^

### Photocatalytic Experimental Setup

Photocatalytic reactions were performed in a glass cell with a top quartz optical window similar to our group's previous publication,^50^ coupled to the same GC analytics (Shimadzu-JAS autosampler + Shimadzu GCMS QP-2020), plus the redundant PST9 in between the reactor and GC sampling line. The main upgrade is the reduction of total volume to 24 mL (height 8 cm, radius 1 cm) maintaining the gas-tight glass to metal standard and the same incident light at 1 sun under AM 1.5G conditions when the cell is filled with 10–12 mL of suspension (when doing *r*_opt_ measurements at less solution volume, the reactor was moved up/down to keep the same irradiance). In addition to regular stirring, the bottom is a fritted glass of porosity 3 or 4.5 (nominal bubble size from 10 to 40 μm), from where the gas enters directly the liquid medium, producing additional means of liquid-gas transport. The inflow of 4.6-purity helium was controlled by a mass flow controller (Bronkhorst low dP series), and at the headspace the overpressure was maintained in continuous flow at 0.5 bar with a back-pressure flow controller (Brooks SLA5820 + 0251 PLC-Power supply). This cell was also jacketed and the temperature of the cooling water was set to 25°C using a thermostat (Lauda Eco RE 415 S). At regular operation helium flow conditions of 10–20 NmL min^−1^, the characteristic times measured for mass transfer coefficients (PST6 trace photoluminescence sensor for dissolved oxygen [Presens]) in our reactor geometries are 30 s and 110 min (50 min under vigorous stirring) with and without a bubbling condition, respectively.^64^ On the other hand, the retention time of the gas in the headspace assuming ideal mixing is calculated and measured as around 1.5 min, which means that in the absence of bubbles the response of the reactor when the light is turned on is mass transfer limited, while with bubbling the response of the reactor is moderately headspace mixing limited. Although literature hints that a more thorough modeling and algorithms are required to calculate the O_2_ that remains dissolved in the liquid during OER experiments in liquid phase and the precise time when the reactor reaches a steady flow,^51^ plugging the numbers of our reactor in such algorithms suggests that the rule of thumb of our previous HER publications in continuous flow that—in the absence of material induction time—after two retention times (in this new reactor ∼2 min), the evolution of oxygen measured by our instrument at the outflow port is equivalent to OER and the dissolved oxygen that remains unmeasured in liquid phase is less than 1% of the total molar fraction of O_2_ measured on-line via headspace. Thus the on-line OER analysis is similar to our previous publication and corresponds to the mass balance of oxygen in the system in steady state:^50^

Equation 1 $$r_{{\text{O}}_{2}}=\frac{F_{\text{ in}}\times \Delta x_{{\text{O}}_{2}\text{,ppm}}\times 10^{-6}}{\left(1-\Delta x_{{\text{O}}_{2}\text{,ppm}}\times 10^{-6}\right)},$$

where *F*_in_ is controlled molar helium (99.996% purity) in flow (mol h^−1^); *r*_O2_ is total OER production rate (10^−6^ μmol h^−1^); Δ*x*_O2,ppm_ is oxygen molar fraction delta at the reactor outlet, measured immediately with PST9 sensor and downstream a splitter with GC-MS-BID. The delta is the raw readout measured after illumination minus the measured baseline in darkness immediately before illumination.

Initially, our reactor design contained liquid oxygen sensors (Clark electrode and photoluminescence sensor) for photocatalytic measurements also, but they were not used further, since both Clark electrodes and photoluminescence sensors in the liquid phase were prone to serious compensation issues at the trace levels of our measurements, and significantly impeded the cleaning of our high-throughput screening regime. Furthermore, after the mass transfer calculations mentioned above, and after verifying that under certain special photocatalytic conditions the increase in liquid oxygen was null, such sensors were proven irrelevant.

### Photocatalytic Activity Screening and *In Situ* RuO_2_ PD Procedure

Suspensions were prepared using 10 mL of Milli-Q water containing 5 mg of sample (P25 or previously activated P25 samples), 10 mM KIO_3_, and the desired volume of TSPP stock. The suspensions were then ultrasonicated for 15 min and finally, in the case of *in situ* RuO_2_ deposition, the desired volume of Ru(III) stock solution was added. The reactor assembled without solution was vacuumed and pressurized two times to displace initial air hold-up, and flowed at the regular controlled helium flow and controlled overpressure until a stable baseline of around 5 ppm of O_2_ in the headspace was obtained. Then, under a mild overpressure, the reactor was quickly opened and the solution was inserted and vacuumed one more time until the same baseline was obtained, and at the time all process values of instrumentation reached the set points. Finally, the light was turned on (Figure S15) together with the first point taken in darkness by the GC-MS-BID autosampler in the case of complementary measurements. The delta of O_2_ was then tracked on-line with the PST9 sensor, and the GC autosampler kept measuring under different routines that minimize the GC analysis time. Additionally, in case of %Ru loading optimization, a Hamilton gas-tight syringe was kept in the reactor ready to inject more Ru precursor repeating light-off/light-on cycles to obtain the OER maxima at each point. After trying this method to obtain optimal %Ru loading *in situ*, the %Ru contents around the maxima were repeated in different vials independently, whose numbers are expressed as error bars in Figure S2. The optimal %Ru loading was identified using a polynomial fit of the data, giving a value of 0.15 Ru(III)/P25 w/w % for PD later used in scaled experiments to obtain *ex situ* deposited optimal samples. In the case of *r*_opt_ experiments of previously Ru-activated P25 optimal samples, consecutively different volumes of suspension were introduced in the reactor and moved accordingly to keep the liquid-gas interface at the same distance from the lamp. For optimal samples for which no full OER rate versus suspension volume curve was reported, two points were tried at 8 mL and 10 mL to ensure that the nominal amount of 10 mL was around the *r*_opt_ value.

### Quantum Efficiency and O_2_ Isotope Counting Measurements

For the labeled water experiment, the long time on-stream (14 h) experiment in Figures 7 and 8 was repeated exactly but at half the suspension volume and using a weight ratio of one-quarter the ^18^O-labeled water (97 atom %, Sigma-Aldrich) to Milli-Q water. The photocatalytic activity procedure was then repeated identically, measuring O_2_ concentrations with the BID detector and tracking molecule counting (MIC) at the MS channels *m*/*z* = 32, *m*/*z* = 34, and *m*/*z* = 36. Such channels' response was integrated around the O_2_ retention time to obtain an additional total O_2_ response using regular MS calibration. Qualitatively, the peaks are shown in Figure S9 to demonstrate that labeled O_2_ species appear only when labeled water was used. Quantitative isotopic distribution was obtained by integrating such peaks, and discounting the ^32^O_2_ mass response in darkness to the one after illumination, after which the percentage of area of channels *m*/*z* = 34 and *m*/*z* = 36 to total O_2_ area was calculated. As MS response is proportional to amount of substance, such area percentage is representative of the actual distribution of O_2_ species (percentage values displayed in Figure 8), and later contrasted to predicted distributions by using a mean field approximation for the case with and without KIE in Table S3. For quantum efficiency experiments, the same reactor was adapted to an alternative design that is jacketless to place a spectrophotometer collecting fiber together with a cosine corrector (Ocean Optics FX-XR1-ES) perpendicular to the outer surface of the reactor. The integration settings were fine-tuned to avoid sensor saturation and maximize signal-to-noise ratio, and the fiber was tried at different angular positions during quick illumination intervals to ensure optimal photon collection. The experiment in Figure 7 was then repeated and the OER activity was recorded to ensure no major deviation from reported trends using the regular reactor while collecting different spectra of side-scattered light with the lamp turned on. After recording, the fiber was moved down 1 mm per step to complete a 32-mm profile. The experimental data that show a good agreement with the optical model predictions are presented in Figure S13.

### Acronyms

AQY: apparent quantum yield

GC-MS-BID: gas chromatography-mass spectrometry-barrier ionization discharge

HT: hydrothermal

IQE: internal quantum efficiency

OER: oxygen evolution reaction

PD: photodeposition

POWS: photocatalytic overall water splitting

SEA: sacrificial electron acceptor

STH: solar-to-hydrogen efficiency

TON: turnover number

TSPP: tetrasodium pyrophosphate

### Indicator Definitions and Estimations

To avoid ambiguity, we have included in this section the typical performance indicator definitions that are used throughout this article and are pertinent to the fields of artificial photosynthesis and photocatalysis. We have gathered the definitions from different literature to more accurately emphasize the ideas and calculations stated in this article and have adapted them to specific reactions of study and flow conditions (many numbers were originally defined for a specific time interval, while in our continuous-flow measurements they are all implicitly a function of time embedded in the calculation of *r*_O2_). Although it is clear that in our previous publications and in the field in general the terms AQY and EQE are most of the time used interchangeably, in the most rigorous sense the term AQY should be used only if information of reaction versus monochromatic light (or narrow wavelength band) is available.^14^^,^^16^^,^^17^^,^^32^^,^^50^ The same distinction applies to IQE and quantum yield (also internal quantum yield). Another strong assumption for the estimations of turnover frequency (TOF) and turnover number (TON) below is the definition that all co-catalyst centers, in this case RuO_2_, are exposed to liquid media, which is indeed the lower limit and refers to the space averaged observed value, because these numbers are intrinsically a function of the local rate of photon absorption and wavelength.

Solar to hydrogen (STH, i.e., overall water splitting):^11^^,^^65^

Equation 2 $$\text{STH}=\;\frac{r_{\text{c}}\times \Delta G_{\text{r}}}{P_{\text{sun}}\times S},$$

where *r*_c_ is production rate of the chemical of interest (mol H_2_ h^−1^); Δ*G*_r_ is Gibbs energy of the reaction (237.000 J mol ^−1^); *P*_sun_, *S* are the energy flux of the sunlight (100 mW cm^−2^) and the area of the reactor.

Apparent quantum yield (AQY, in photocatalytic OER):^13^^,^^15^^,^^17^

Equation 3 $$\text{AQY}\left(\lambda \right)\%=\;\frac{4\times r_{{\text{O}}_{2}}}{I_{0}\left(\lambda \right)},$$

where *I*_0_(λ) is incident photon rate at a specified wavelength *λ* (i.e., 10^−6^ μE h^−1^).

Quantum yield (Φ, in photocatalytic OER):^13^^,^^15^^,^^17^

Equation 4a $$\Phi \left(\lambda \right)\%=\;\frac{4\times r_{O_{2}}}{I_{abs}\left(\lambda \right)},$$

where *I*_abs_(*λ*) is total absorbed photon rate at a specified wavelength *λ* (i.e., 10^−6^ μE h^−1^).

Photonic efficiency (ξ_e_):^13^^,^^15^^,^^17^

Equation 4b $$\xi_{e}\left(\lambda_{i},\lambda_{j}\right)\%=\;\frac{4\times r_{O_{2}}}{I_{0}\left(\lambda_{i},\lambda_{j}\right)},$$

where *I*_0_(*λ*_*i*_, *λ*_*j*_) is incident photon rate at a specified wavelength range between *λ*_*i*_ and *λ*_*j*_ (i.e., 10^−6^ μE h^−1^).

Quantum efficiency (QE):^13^^,^^15^^,^^17^

Equation 5 $$\text{QE}\left(\lambda_{i},\lambda_{j}\right)\%=\;\frac{4\times r_{{\text{O}}_{2}}}{I_{\text{abs}}\left(\lambda_{i},\lambda_{j}\right)},$$

where *I*_abs_(*λ*_*i*_, *λ*_*j*_) is absorbed photon rate at a specified wavelength range between *λ*_*i*_ and *λ*_*j*_.

Turnover frequency (TOF_avg_) and Turnover number (TON_avg_):^20^^,^^50^

Equation 6 $${\text{TON}}_{\text{avg}}=\int_{t=0}^{t_{\text{f}}}r_{{\text{O}}_{2}}\left(t\right)dt/\text{μmol of Ru }\left(\text{ICP}\right),$$

Equation 7 $${\text{TOF}}_{\text{avg}}=r_{{\text{O}}_{2}}/\text{μmol of Ru }\left(\text{ICP}\right),$$

Relative photonic efficiency (ξ′_e_, using OER benchmark):

Equation 8 $${\text{ξ}}_{\text{e}}^{\prime }\left(\lambda_{i},\lambda_{j}\right)=\;\frac{r_{\text{opt},x}}{I_{0,x}\left(\lambda_{i},\lambda_{j}\right)}/\frac{r_{\text{opt},0}}{I_{0}},$$

where *I*_0_ is incident photon rate for OER benchmark at near AM 1.5G conditions; *I*_0,*x*_(*λ*_*i*_, *λ*_*j*_) is incident photon rate for a material *x* to be normalized, at a specified wavelength range between *λ*_*i*_ and *λ*_*j*_, equal to *I*_0_ if AM 1.5G is also used for material *x*; *r*_opt,0_ is optimal photocatalytic OER production rate of OER benchmark (10^−6^ μmol h^−1^), at a condition whereby adding more suspension volume at constant *I*_0_ at the liquid-gas interface produces no further increase of measured OER; *r*_opt,*x*_ is optimal photocatalytic OER production rate of material *x* to be normalized (10^−6^ μmol h^−1^) at a condition whereby adding more suspension volume (or other forms of catalyst load) at constant *I*_0,*x*_(*λ*_*i*_, *λ*_*j*_) at the liquid-gas interface produces no further increase of measured OER.

### Optical Models

To simulate the light propagation in our reactor under the same conditions of the photocatalysis experiment, we employ a theoretical approach based on the Monte Carlo method together with scattering Mie theory similar to that reported by some of us in previous works.^66^, ^67^, ^68^, ^69^ The model considered here consists of a cylindrical glass reactor with a quartz window (mimicking the one used in the experiment) that contains the TiO_2_ nanoparticle suspension under study (Figure S12), in which each material considered (glass, quartz, water, and TiO_2_) is described by the complex refractive index:

Equation 9 $$\tilde{n}\left(\lambda \right)=n\left(\lambda \right)+ik\left(\lambda \right),$$

where *λ* corresponds to the incident wavelength. For the nanoparticle suspension, the volumetric effective medium approximation is considered; it is at this point where the TiO_2_ concentration of the suspension under study is introduced into the model together with the agglomerate size (Figure S10). This approach allows us to follow the trajectory of each photon individually (Figure S11). These trajectories are described by the Fresnel coefficients at the interfaces and by the Mie theory when the photon interacts with TiO_2_ nanoparticles.^66^ By monitoring the trajectories of all the photons (Figure S11), we can sketch the reflectance, transmittance, and absorptance spectra of the system under study (Figure 9). Furthermore, the parasitic absorption due to the solvent or the walls of the reactor itself can also be evaluated. Moreover, the information of the trajectories of each photon individually allows us to know the spatial and spectral distributions of the absorption processes (Figure S14). To ensure the convergence of all these distributions, the system is pumped with 10^6^ photons for each wavelength.

## Supplemental Information

Document S1. Figures S1–S16 and Tables S1–S3

Document S2. Article plus Supplemental Information

## Footnotes

Supplemental Information can be found online at https://doi.org/10.1016/j.matt.2020.07.021.

## References

[1] Walsh B., Ciais P., Janssens I.A., Peñuelas J., Riahi K., Rydzak F., van Vuuren D.P., Obersteiner M. (2017). Pathways for balancing CO_2_ emissions and sinks. Nat. Commun..

[2] Peters G.P., Andrew R.M., Canadell J.G., Friedlingstein P., Jackson R.B., Korsbakken J.I., Le Quéré C., Peregon A. (2020). Carbon dioxide emissions continue to grow amidst slowly emerging climate policies. Nat. Clim. Chang..

[3] Bosetti V., Weber E., Berger L., Budescu D.V., Liu N., Tavoni M. (2017). COP21 climate negotiators’ responses to climate model forecasts. Nat. Clim. Chang..

[4] Osterloh F.E. (2017). Photocatalysis versus photosynthesis : a sensitivity analysis of devices for solar energy. ACS Energy Lett..

[5] Wang Q., Domen K. (2020). Particulate photocatalysts for light-driven water splitting: mechanisms, challenges, and design strategies. Chem. Rev..

[6] Chen X., Shen S., Guo L., Mao S.S. (2010). Semiconductor-based photocatalytic hydrogen generation. Chem. Rev..

[7] Roger I., Shipman M.A., Symes M.D. (2017). Earth-abundant catalysts for electrochemical and photoelectrochemical water splitting. Nat. Rev. Chem..

[8] Chen S., Takata T., Domen K. (2017). Particulate photocatalysts for overall water splitting. Nat. Publ. Gr..

[9] Ma Y., Wang X., Jia Y., Chen X., Han H., Li C. (2014). Titanium dioxide-based nanomaterials for photocatalytic fuel generations. Chem. Rev..

[10] Linsebigler A.L., Lu G., Yates J.T. (1995). Photocatalysis on TiOn surfaces: principles, mechanisms, and selected results. Chem. Rev..

[11] Goto Y., Hisatomi T., Wang Q., Higashi T., Ishikiriyama K., Maeda T., Sakata Y., Okunaka S., Tokudome H., Katayama M. (2018). A particulate photocatalyst water-splitting panel for large-scale solar hydrogen generation. Joule.

[12] Wang Q., Hisatomi T., Jia Q., Tokudome H., Zhong M., Wang C., Pan Z., Takata T., Nakabayashi M., Shibata N. (2016). Scalable water splitting on particulate photocatalyst sheets with a solar-to-hydrogen energy conversion efficiency exceeding 1%. Nat. Mater..

[13] Qureshi M., Takanabe K. (2017). Insights on measuring and reporting heterogeneous photocatalysis: efficiency definitions and setup examples. Chem. Mater..

[14] Braslavsky S.E., Braun A.M., Cassano A.E., Emeline A.V., Litter M.I., Palmisano L., Parmon V.N., Serpone N. (2011). Glossary of terms used in photocatalysis and radiation catalysis (IUPAC Recommendations 2011). Pure Appl. Chem..

[15] Kisch H., Bahnemann D. (2015). Best practice in photocatalysis: comparing rates or apparent quantum yields?. J. Phys. Chem. Lett..

[16] Cabrera M.I., Alfano O.M., Cassano A.E. (1994). Novel reactor for photocatalytic kinetic studies. Ind. Eng. Chem. Res..

[17] Serpone N. (1997). Relative photonic efficiencies and quantum yields in heterogeneous photocatalysis. J. Photochem. Photobiol. A Chem..

[18] Grewe T., Meggouh M., Tüysüz H. (2016). Nanocatalysts for solar water splitting and a perspective on hydrogen economy. Chem. Asian J..

[19] Schlögl R. (2015). Heterogeneous catalysis. Angew. Chem. Int. Ed..

[20] Wachs I.E., Phivilay S.P., Roberts C.A. (2013). Reporting of reactivity for heterogeneous photocatalysis. ACS Catal..

[21] Emeline A.V., Ryabchuk V.K., Serpone N., May R.V. (2005). Dogmas and misconceptions in heterogeneous photocatalysis. Some enlightened reflections. J. Phys. Chem. B.

[22] Emeline A.V., Zhang X., Jin M., Murakami T., Fujishima A. (2006). Application of a “black body” like reactor for measurements of quantum yields of photochemical reactions in heterogeneous systems. J. Phys. Chem. B.

[23] Kunz L.Y., Diroll B.T., Wrasman C.J., Riscoe A.R., Majumdar A., Cargnello M. (2019). Artificial inflation of apparent photocatalytic activity induced by catalyst-mass-normalization and a method to fairly compare heterojunction systems. Energy Environ. Sci..

[24] Megatif L., Dillert R., Bahnemann D.W. (2019). Reaction rate study of the photocatalytic degradation of dichloroacetic acid in a black body reactor. Catalysts.

[25] Megatif L., Dillert R., Bahnemann D.W. (2019). Determination of the quantum yield of a heterogeneous photocatalytic reaction employing a black body photoreactor. Catal. Today.

[26] Ollis D.F. (2005). Kinetic disguises in heterogeneous photocatalysis. Top. Catal..

[27] Wang Z., Li C., Domen K. (2019). Recent developments in heterogeneous photocatalysts for solar-driven overall water splitting. Chem. Soc. Rev..

[28] ISO (International Organization for Standardization) (2016). ISO 22197-1. Fine Ceramics (Advanced Ceramics, Advanced Technical Ceramics)—Test Method for Air-Purification Performance of Semiconducting Photocatalytic Materials—Part 1: Removal of Nitric Oxide. https://www.iso.org/standard/65416.html.

[29] ISO (International Organization for Standardization) (2019). ISO 22197-2. Fine Ceramics (Advanced Ceramics, Advanced Technical Ceramics)—Test Method for Air-Purification Performance of Semiconducting Photocatalytic Materials—Part 2: Removal of Acetaldehyde. https://www.iso.org/standard/72347.html.

[30] Pougin A., Dilla M., Strunk J. (2016). Identification and exclusion of intermediates of photocatalytic CO_2_ reduction on TiO_2_ under conditions of highest purity. Phys. Chem. Chem. Phys..

[31] Dilla M., Jakubowski A., Strunk J., Ristig S., Strunk J., Schlögl R. (2019). The fate of O_2_ in photocatalytic CO_2_ reduction on TiO_2_ under conditions of highest purity. Phys. Chem. Chem. Phys..

[32] Dilla M., Moustakas N.G., Becerikli A.E., Peppel T., Springer A., Schlo R., Strunk J., Ristig S. (2019). Judging the feasibility of TiO_2_ as photocatalyst for chemical energy conversion by quantitative reactivity determinants. Phys. Chem. Chem. Phys..

[33] Dilla M., Schlçgl R., Strunk J. (2017). Photocatalytic CO_2_ reduction under continuous flow high-purity conditions: quantitative evaluation of CH_4_ formation in the steady-state. ChemCatChem.

[34] Huang L., Li R., Chong R., Liu G., Han J., Li C. (2014). Cl^-^ making overall water splitting possible on TiO_2_-based photocatalysts. Catal. Sci. Technol..

[35] Hashimoto K., Irie H., Fujishima A. (2006). TiO_2_ photocatalysis: a historical overview and future prospects. Jpn. J. Appl. Phys..

[36] Li R., Weng Y., Zhou X., Wang X., Mi Y., Chong R., Han H., Li C. (2015). Achieving overall water splitting using titanium dioxide-based photocatalysts of different phases. Energy Environ. Sci..

[37] Wenderich K., Mul G. (2016). Methods, mechanism, and applications of photodeposition in Photocatalysis: a review. Chem. Rev..

[38] Mills A., Duckmanton P.A., Reglinski J. (2010). A simple, novel method for preparing an effective water oxidation catalyst. Chem. Commun..

[39] Mi S.Y., Liu Y.X., Wang W.D. (2016). Photo-depositing Ru and RuO_2_ on anatase TiO_2_ nanosheets as Co-catalysts for photocatalytic O_2_ evolution from water oxidation. Chin. J. Chem. Phys..

[40] Jiao Y., Jiang H., Chen F. (2014). RuO_2_/TiO_2_/Pt ternary photocatalysts with epitaxial heterojunction and their application in CO oxidation. ACS Catal..

[41] Xiang G., Shi X., Wu Y., Zhuang J., Wang X. (2012). Size effects in atomic-level epitaxial redistribution process of RuO_2_ over TiO_2_. Sci. Rep..

[42] Teleki A., Wengeler R., Wengeler L., Nirschl H., Pratsinis S.E. (2008). Distinguishing between aggregates and agglomerates of flame-made TiO_2_ by high-pressure dispersion. Powder Technol..

[43] Suttiponparnit K., Jiang J., Sahu M., Suvachittanont S. (2011). Role of surface area, primary particle size, and crystal phase on titanium dioxide nanoparticle dispersion properties. Nanoscale Res. Lett..

[44] Jiang J., Oberdörster G., Biswas P. (2009). Characterization of size, surface charge, and agglomeration state of nanoparticle dispersions for toxicological studies. J. Nanoparticle Res..

[45] Zhao D., Chen C., Wang Y., Ji H., Ma W., Zang L., Zhao J. (2008). Surface modification of TiO_2_ by phosphate: effect on photocatalytic activity and mechanism implication. J. Phys. Chem. C.

[46] Ajmal A., Majeed I., Malik R.N., Idriss H., Nadeem M.A. (2014). Principles and mechanisms of photocatalytic dye degradation on TiO_2_ based photocatalysts: a comparative overview. RSC Adv..

[47] Persson K. (2016). Materials Data on RuO2 (SG:136) by Materials Project.

[48] Persson K. (2016). Materials Data on Ru (SG:194) by Materials Project.

[49] Povar I., Spinu O. (2016). Ruthenium redox equilibria: 3. Pourbaix diagrams for the systems Ru-H_2_O and Ru-Cl^-^-H_2_O. J. Electrochem. Sci. Eng..

[50] Biswal B.P., Vignolo-González H.A., Banerjee T., Grunenberg L., Savasci G., Gottschling K., Nuss J., Ochsenfeld C., Lotsch B.V. (2019). Sustained solar H_2_ evolution from a thiazolo[5,4-d]thiazole-bridged covalent organic framework and nickel-thiolate cluster in water. J. Am. Chem. Soc..

[51] Hernández S., Bensaid S., Armandi M., Sacco A., Chiodoni A., Bonelli B., Garrone E., Pirri C.F., Saracco G. (2014). A new method for studying activity and reaction kinetics of photocatalytic water oxidation systems using a bubbling reactor. Chem. Eng. J..

[52] Limburg B., Bouwman E., Bonnet S. (2016). Rate and stability of photocatalytic water oxidation using [Ru(bpy)3]^2+^ as photosensitizer. ACS Catal..

[53] Schneider J., Bahnemann D.W. (2013). Undesired role of sacrificial reagents in photocatalysis. J. Phys. Chem. Lett..

[54] Abe R., Sayama K., Domen K., Arakawa H. (2001). A new type of water splitting system composed of two different TiO_2_ photocatalysts (anatase, rutile) and a IO_3_^-^/I^-^ shuttle redox mediator. Chem. Phys. Lett..

[55] Abe R., Sayama K., Arakawa H. (2003). Significant effect of iodide addition on water splitting into H_2_ and O_2_ over Pt-loaded TiO_2_ photocatalyst: suppression of backward reaction. Chem. Phys. Lett..

[56] Bui T.D., Yagi E., Harada T., Ikeda S., Matsumura M. (2012). Isotope tracing study on oxidation of water on photoirradiated TiO_2_ particles. Appl. Catal. B Environ..

[57] Ding Q., Liu Y., Chen T., Wang X., Feng Z., Wang X., Dupuis M., Li C. (2020). Unravelling the water oxidation mechanism on NaTaO_3_-based photocatalyst. J. Mater. Chem. A.

[58] Miyoshi A., Nishioka S., Maeda K. (2018). Water splitting on rutile TiO_2_-based photocatalysts. Chem. Eur. J..

[59] Valdés Á., Qu Z.W., Kroes G.J., Rossmeisl J., Nørskov J.K. (2008). Oxidation and photo-oxidation of water on TiO_2_ surface. J. Phys. Chem. C.

[60] Banerjee T., Haase F., Savasci G., Gottschling K., Ochsenfeld C., Lotsch B.V. (2017). Single-site photocatalytic H_2_ evolution from covalent organic frameworks with molecular cobaloxime Co-catalysts. J. Am. Chem. Soc..

[61] Laha S., Lee Y., Podjaski F., Weber D., Duppel V., Schoop L.M., Pielnhofer F., Scheurer C., Müller K., Starke U. (2019). Ruthenium oxide nanosheets for enhanced oxygen evolution catalysis in acidic medium. Adv. Energy Mater..

[62] Wang M., Nie B., Yee K.K., Bian H., Lee C., Lee H.K., Zheng B., Lu J., Luo L., Li Y.Y. (2016). Low-temperature fabrication of brown TiO_2_ with enhanced photocatalytic activities under visible light. Chem. Commun..

[63] Morgan D.J. (2015). Resolving ruthenium: XPS studies of common ruthenium materials. Surf. Interf. Anal..

[64] Lewis R.S., Deen W.M. (1994). Kinetics of the reaction of nitric oxide with oxygen in aqueous solutions. Chem. Res. Toxicol..

[65] Hisatomi T., Domen K. (2017). Introductory lecture: sunlight-driven water splitting and carbon dioxide reduction by heterogeneous semiconductor systems as key processes in artificial photosynthesis. Faraday Discuss..

[66] Li Y., Carretero-Palacios S., Yoo K., Kim J.H., Jiménez-Solano A., Lee C.-H., Míguez H., Ko M.J. (2016). Maximized performance of dye solar cells on plastic: a combined theoretical and experimental optimization approach. Energy Environ. Sci..

[67] Sudiarta I.W., Chylek P. (2001). Mie-scattering formalism for spherical particles embedded in an absorbing medium. J. Opt. Soc. Am. A..

[68] Miranda-Muñoz J.M., Esteso V., Jiménez-Solano A., Lozano G., Míguez H. (2020). Finite size effects on light propagation throughout random media: relation between optical properties and scattering event statistics. Adv. Opt. Mater..

[69] Miranda-Muñoz J.M., Carretero-Palacios S., Jiménez-Solano A., Li Y., Lozano G., Míguez H. (2016). Efficient bifacial dye-sensitized solar cells through disorder by design. J. Mater. Chem. A.

