# Supplementary material for: Toward Standardized Photocatalytic Oxygen Evolution Rates Using RuO2@TiO2 as a Benchmark
Source: Matter. 2020 Aug 5;3(2):464–86. doi: 10.1016/j.matt.2020.07.021 (PMC7418450; doi:10.1016/j.matt.2020.07.021)
Supplement: Document S1. Figures S1–S16 and Tables S1–S3 [file mmc1.pdf]

**Matter, Volume *3***

## Supplemental Information

# Toward Standardized Photocatalytic Oxygen Evolution Rates Using $RuO_2$@$TiO_2$ as a Benchmark

**Hugo A. Vignolo-González, Sourav Laha, Alberto Jiménez-Solano, Takayoshi Oshima, Viola Duppel, Peter Schützendübe, and Bettina V. Lotsch**

## SUPPLEMENTAL INFORMATION

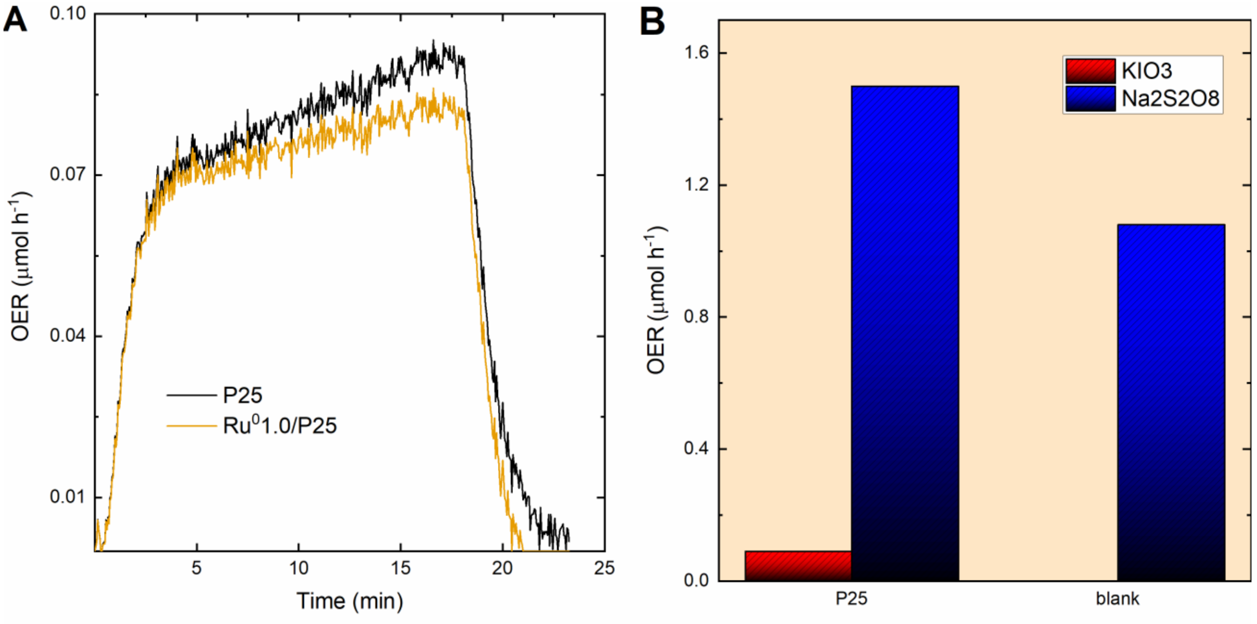


**Figure S1. OER blanks. (A)** P25 and metallic Ru XPS reference ($Ru^0$1.0/P25 – PD) background at typical reaction conditions. Suspension conditions: 5 mg sample, 10 mL water (10 mM $KIO_3$, 0-1 mM TSPP). Other blanks not included due to response below Lower Detection Limit (LDL): 10 mM $KIO_3$ + light; water + light, 10 mM $KIO_3$ + 5 mg nanosilica + light, $Ru^0$1.0/P25 – PD + SEA + TSPP + no light (dark or cut-off filter 420 nm). P25 controls during different deposition process without metal precursor were recovered at identical conditions as active samples and tried for activity testing and characterization, with identical response to fresh P25. **(B)** SEA OER response to illumination in the presence (P25) and absence (blank) of photoabsorber. $KIO_3$ (10 mM) condition is AM 1.5G illumination, 10 mL suspension with a concentration of and 1 mM TSPP as dispersing agent for both blank and photoabsorber, and suspension density of 0.5 mg $mL^{-1}$ for P25. $Na_2S_2O_8$ (20 mM) condition is a Xe lamp full arc illumination at 150 mW $cm^{-2}$, 10 mL suspension with no dispersing agent for both blank and photoabsorber, and suspension density of 1 mg $mL^{-1}$ for P25.

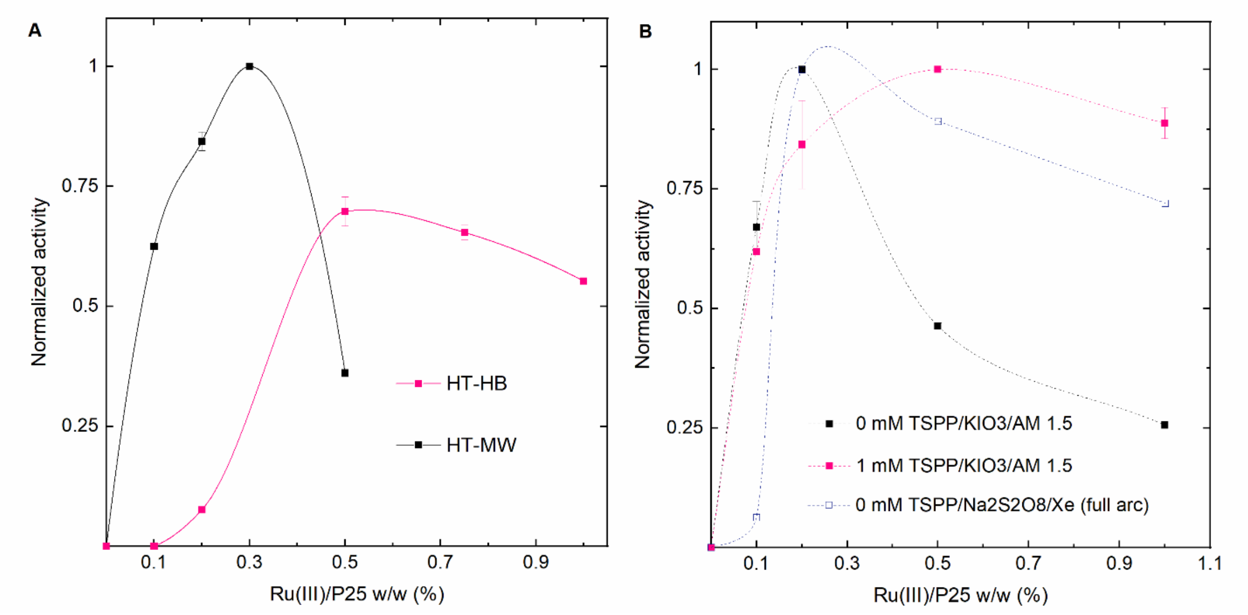


**Figure S2. Ru loading optimization curves. (A)** Ex-situ HT deposited P25/$RuO_2$ samples OER activity testing. Suspension conditions: 5 mg sample, 5 mL water (10 mM $KIO_3$, 0 mM TSPP), and full arc Xe lamp illumination (Data points are presented as mean ± SEM of duplicated batches). **(B)** In-situ Ru(III) PD+OER in activity testing setup vs P25. Suspension conditions: 5 mg sample, 10 mL water (10 mM $KIO_3$, 0-1 mM TSPP), and AM 1.5G or full arc Xe lamp (~ 150 mW $cm^{-2}$) illumination.

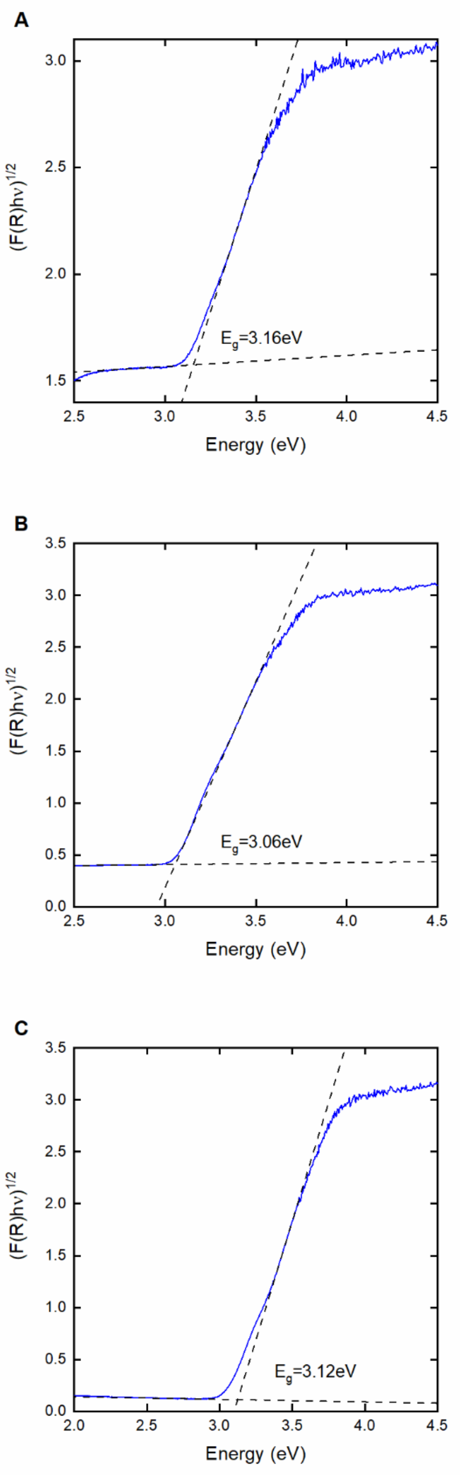


**Figure S3. UV-visible spectra of P25 deposited with $RuO_2$.** Tauc plots obtained from diffuse reflectance measurements of samples **(A)** Ru1.0/P25-HT-HB, **(B)** Ru0.15/P25-PD*, and **(C)** P25 control.

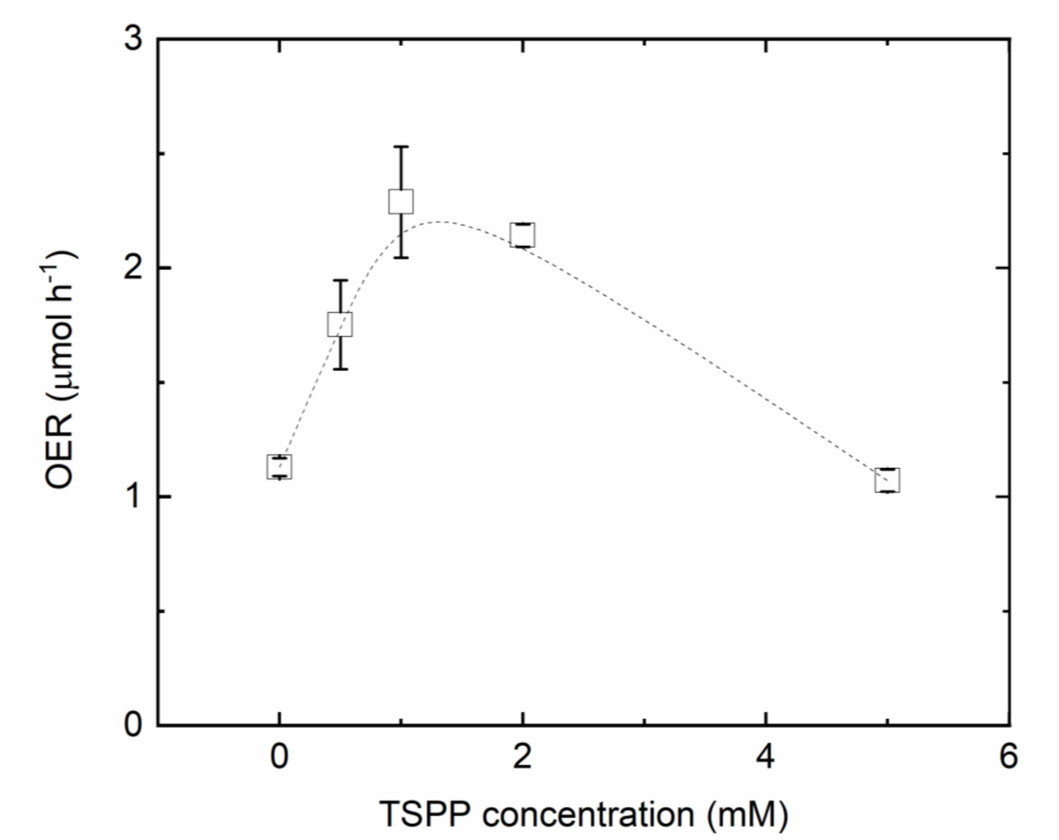


**Figure S4. Dispersant (TSPP) optimization.** P25 and reaction media vials were prepared at different levels of TSPP around the optimal precursor Ru loading obtained at 1 mM TSPP (0.5% w/w Ru(III)/P25, 10 mM KIO3, {0; 0.5; 1; 5} mM TSPP) (Data points are presented as mean ± SEM of duplicated measurements).

**Table S1. ICP-OES Ruthenium contents vs nominal contents.** Nominal values are amount of Ru precursor during deposition step. In descending order the samples correspond to optimal ex-situ PD using dispersing agent (1 mM TSPP); optimal ex-situ PD using no dispersing agent; $RuO_2$ XPS; and metallic Ru XPS reference. P25 controls displayed no traces of precursor, sacrificial, or dispersant agent. Standard deviation (σ) is presented in % of the mean ICP value.

| Sample name | nominal Ru %w/w content | ICP mean % | σ/mean % | Yield % |
|---|---|---|---|---|
| **0.15Ru/P25 – PD*** | 0.15 | 0.08 | 1.22 | 55 |
| **0.15Ru/P25 - PD** | 0.15 | 0.08 | 4.15 | 52 |
| **Ru1.0/P25-HT-HB** | 1.0 | 0.43 | 0.12 | 43 |
| **$Ru^0$1.0/P25-PD** | 1.0 | 0.39 | 0.77 | 39 |

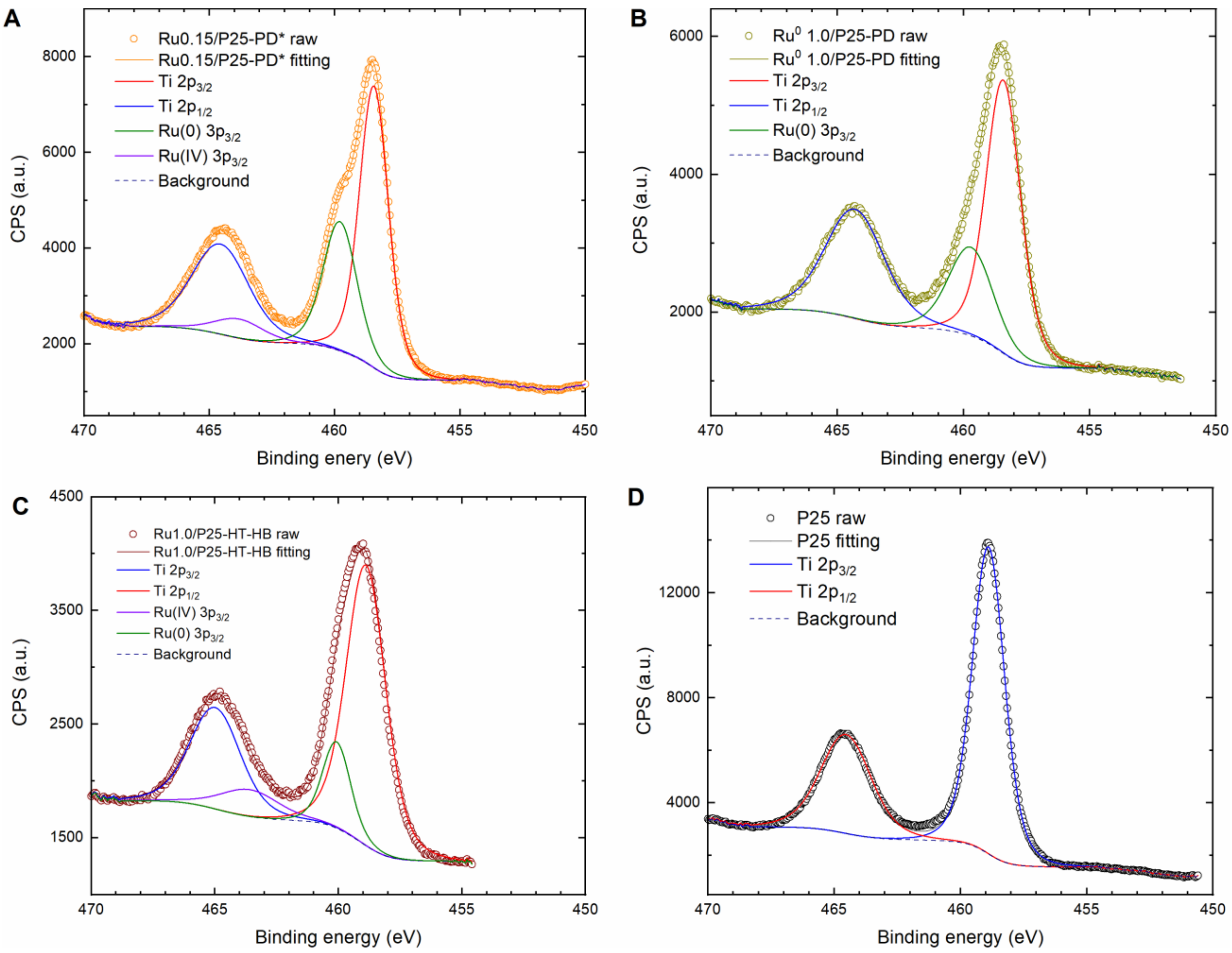


**Figure S5. Full XPS spectra of optimal PD sample, and $RuO_2$ and metallic Ru references. (A)** Ru0.15/P25-PD*, (**B)** $Ru^0$1.0/P25-PD, **(C)** Ru1.0/P25-HT-HB, **(D)** P25 control.

**Table S2. XPS quantitative analysis.** Calculated from Figure S5 for Ru containing samples.

| | Ru0.15/P25 – PD* | Ru1.0/P25 - HT-HB | $Ru^0$1.0/P25 - PD |
|---|---|---|---|
| **Ru 3p I (position) (eV)** | 459.7 | 460.0 | 459.8 |
| **Ru 3p I (area) (a.u.)** | 4922 | 1160 | 3059 |
| **Ru 3p II (position) (eV)** | 463.7 | 463.5 | -- |
| **Ru 3p II (area) (a.u.)** | 1064 | 758 | -- |

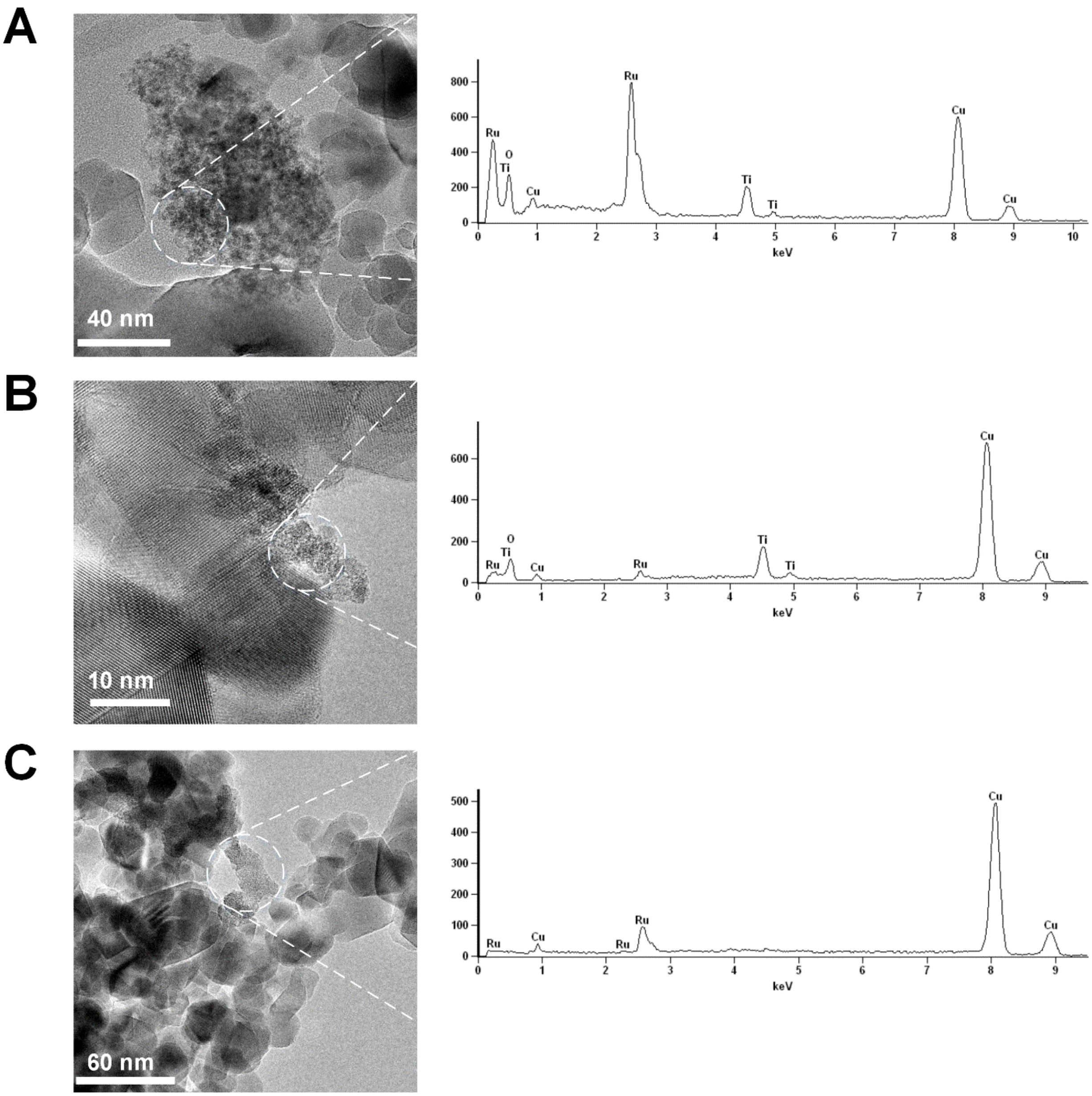


**Figure S6. TEM-EDX raw spectra. (A)** Ru$^0$ 1.0/P25-PD (metallic Ru XPS reference, scale bar: 40 nm), **(B)** Ru1.0/P25-HT-HB ($RuO_2$ XPS reference, scale bar: 10 nm), **(C)** Ru0.15/P25-PD (amorphous metallic Ru zone, scale bar: 60 nm).

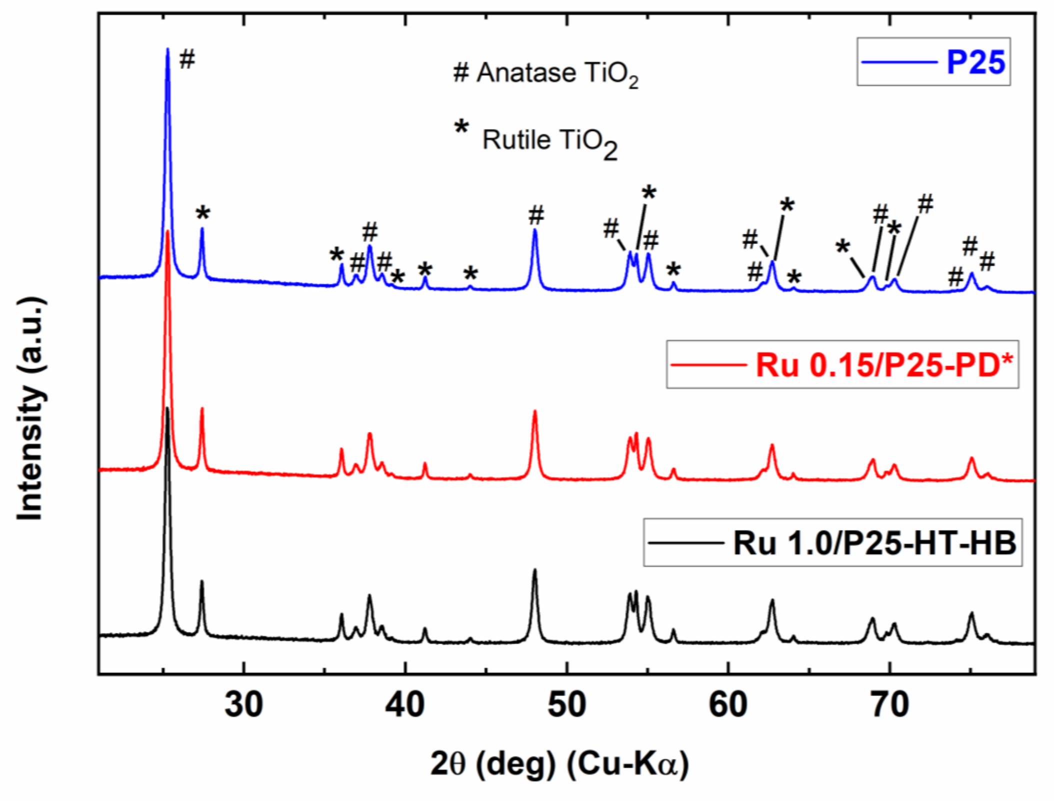


**Figure S7. PXRD pattern of P25 deposited with $RuO_2$.** X-ray powder diffractogram of samples Ru1.0/P25-HT-HB, Ru0.15/P25-PD*, and P25 control. Both anatase and rutile $TiO_2$ peaks are marked for P25.

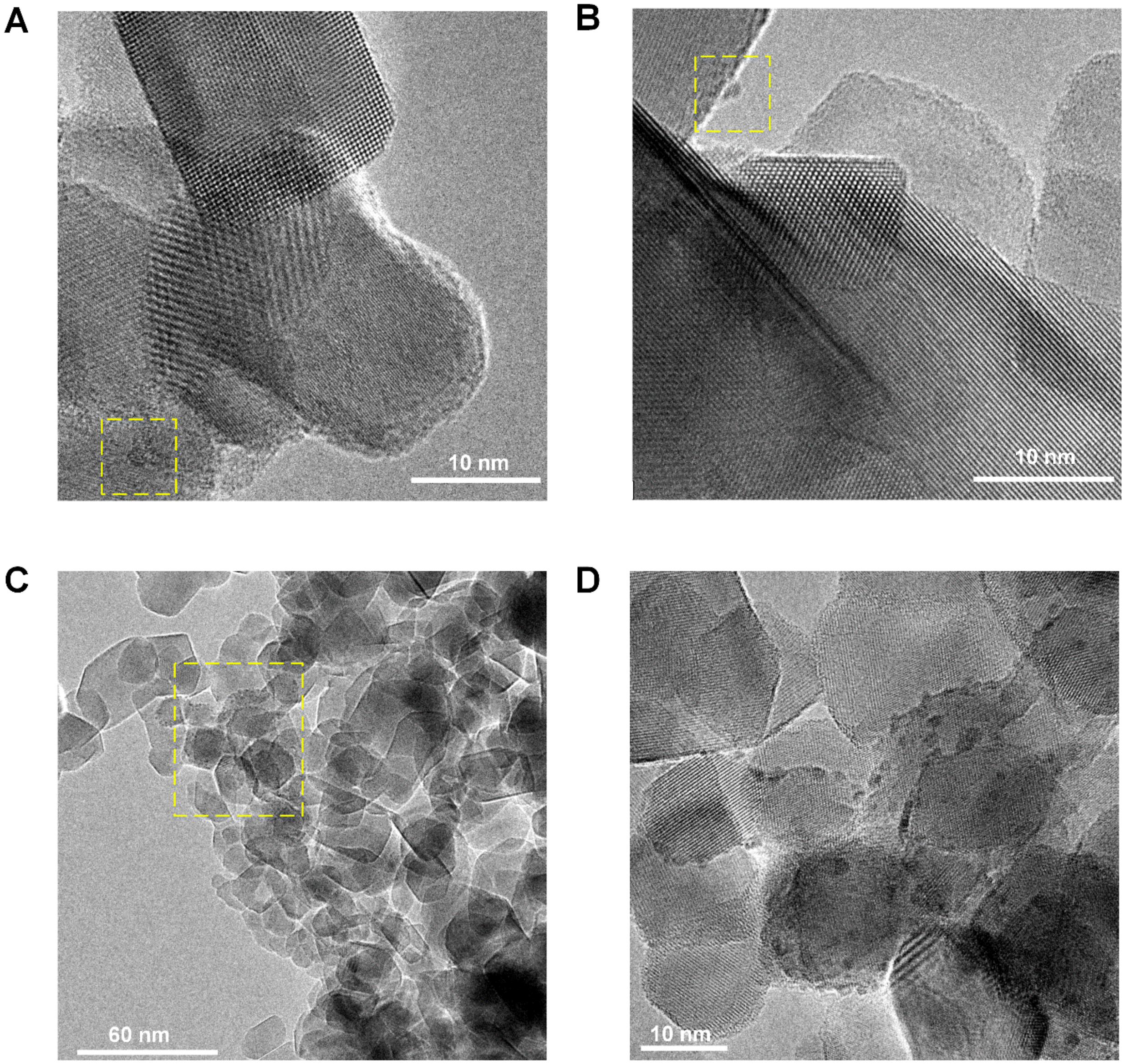


**Figure S8. TEM images of Ru species zones on P25. (A), (B)** After ex-situ PD at 0.5 mg $mL^{-1}$, AM 1.5 filter, and 1 mM TSSP (Ru0.15/P25-PD*, scale bar: 10 nm). **(C), (D)** After ex-situ PD at 0.5 mg $mL^{-1}$, full arc illumination, and no TSSP (Ru0.15/P25-PD, scale bar: 60 and 10 nm respectively).

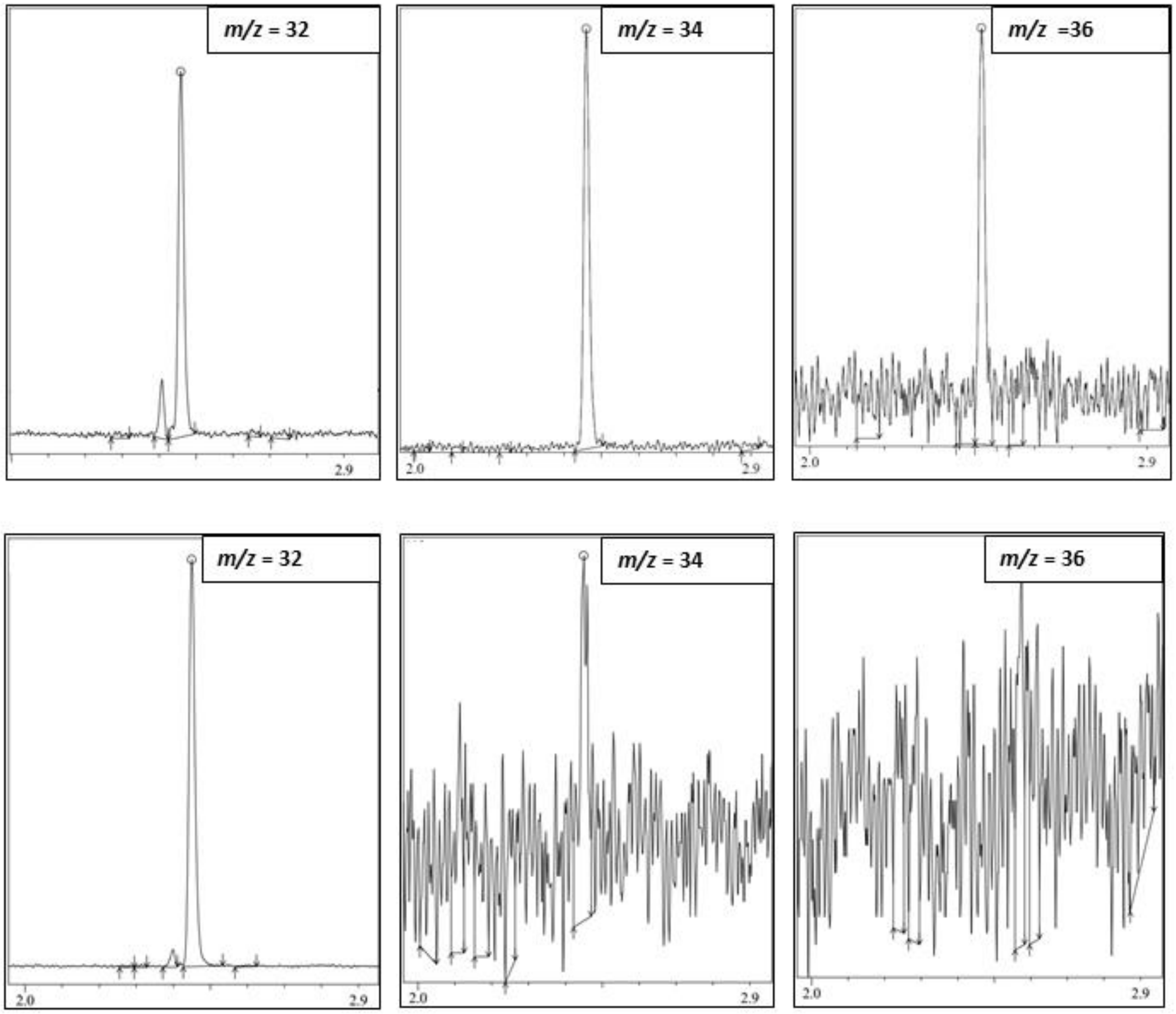


**Figure S9. Raw MS response around $O_2$ GC retention time.** *m*/*z* = 32 ($^{32}O_2$), *m*/*z* = 34 ($^{16}O^{18}O$) and *m*/*z* = 36 ($^{36}O_2$) counting peaks at $O_2$ retention time (2.45 min) obtained after 12 minutes of sample illumination, Top row: when using a ratio of 1/3 in weight of $H_2^{18}O$ to $H_2O$ (complement BID quantification), Bottom row: under normal reaction conditions as in Figure 7. Integration of peaks is proportional to analyte mass and calibration of MS and quantitative BID detector is considered for ppm calculation. Isotopic distribution is obtained by % of ΔA of the species to total integrated $O_2$ area (A) of channels *m*/*z* = 32, 34, 36, where ΔA is obtained subtracting areas measured in dark background.

**Table S3. Ratios of labeled $O_2$ species (*m/z* = 34 and *m/z* = 36) to $O_2$ normal mass (*m/z* = 32).** Theoretical distribution without Kinetic Isotope Effect (Column: Theoretical no KIE) is predicted using only the mean field approximation (MFA) in molar fraction of labeled water molecules (no KIE). If reaction rate limiting step (RLS) is at the bottleneck predicted by reference literature[59], using a MFA and the ratio $k_{RLS1}/k_{RLS2}$~$(m_2/m_1)^{½}$ for reaction probability correction, a KIE distribution is obtained (Column: Theoretical with KIE). Error columns are calculated in % relative to experimental average observation.

| $x/^{32}O_2$ | Experimental (average) | Theoretical No KIE | Theoretical KIE | Error % (no KIE) | Error % (KIE) |
|---|---|---|---|---|---|
| $^{18}O^{16}O$ | 0.54 | 0.67 | 0.55 | 18.9 | 1.2 |
| $^{36}O_2$ | 0.08 | 0.11 | 0.07 | 31.4 | -1.9 |

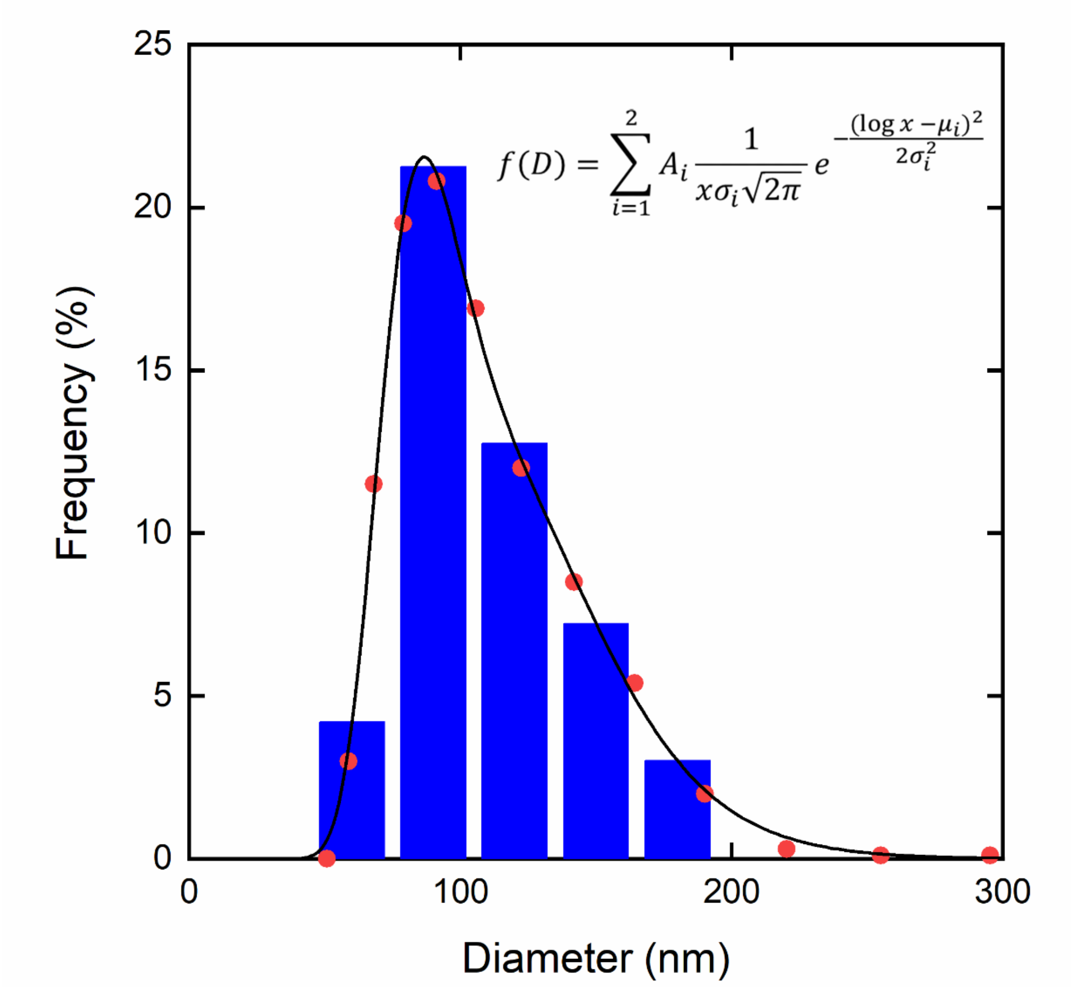


**Figure S10. Redefinition of Dynamic light scattering (DLS) particle size distributions (dots) of Ru0.15/P25-PD* nanoparticles.** The solid line is the fit to the double log-normal size distribution *f*(D) of inset equation. The histogram bars represent the sizes and frequencies used as input for the Monte Carlo calculation of light propagation in the suspension.

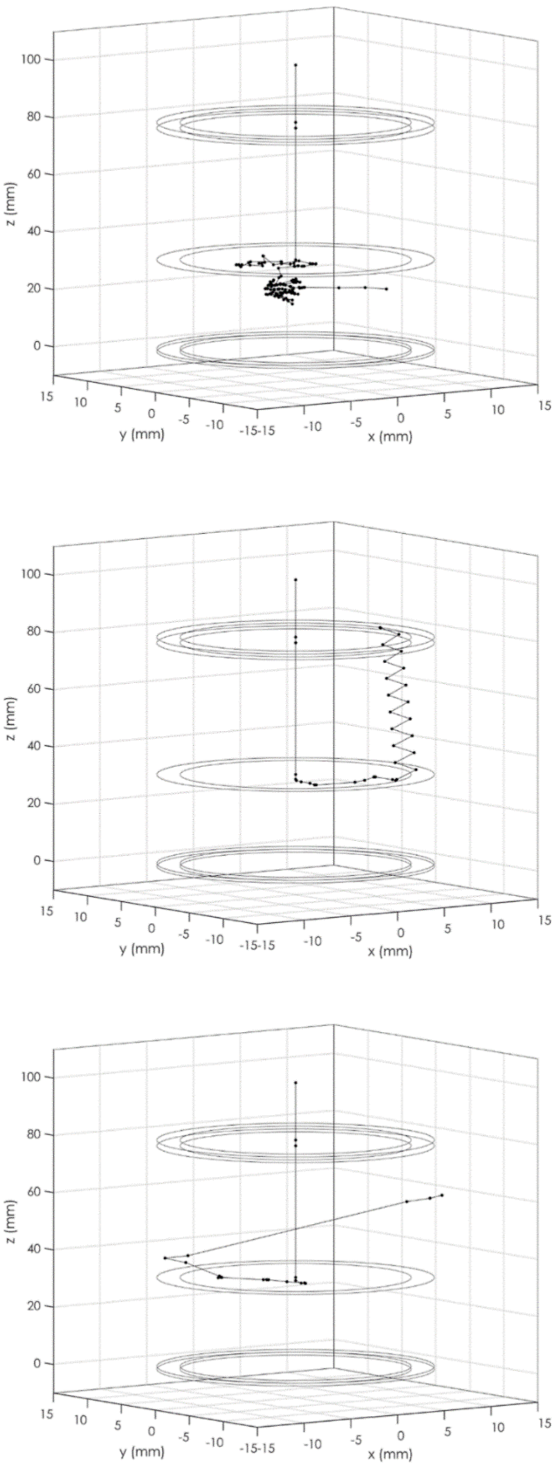


**Figure S11. Photon trajectories in optical modeling. From top to bottom, absorbed, back-scattered and side-scattered.** Representation of 80 mm reactor filled with 10 mL of the considered suspension integrating scattering centers with size distribution shown in Figure S10 and a concentration of 0.01586 vol% of nanoparticles. The photon enters the reactor at z = 80 mm and the interface air-suspension is located at z = 32 mm.

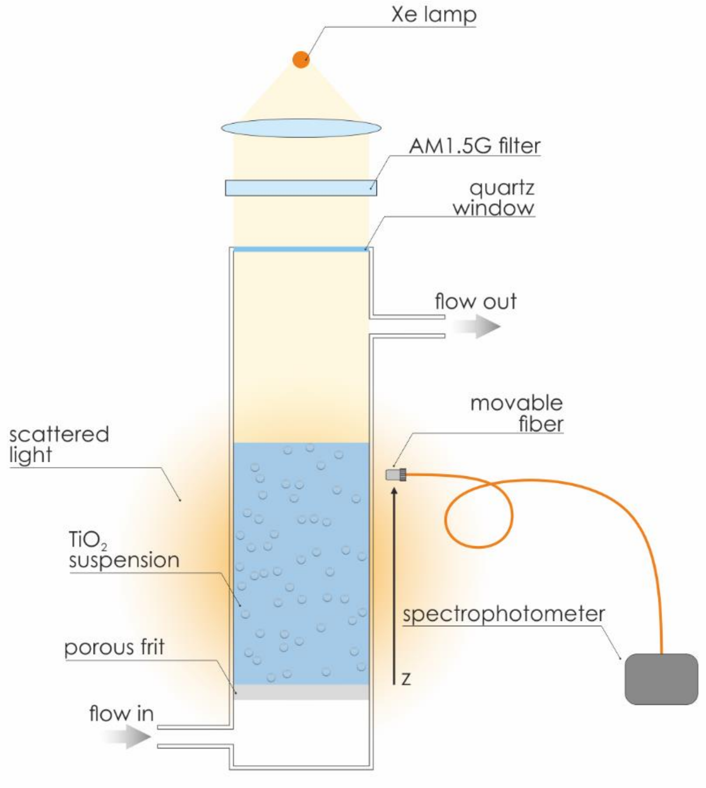


**Figure S12. Quantum efficiency experimental setup.**

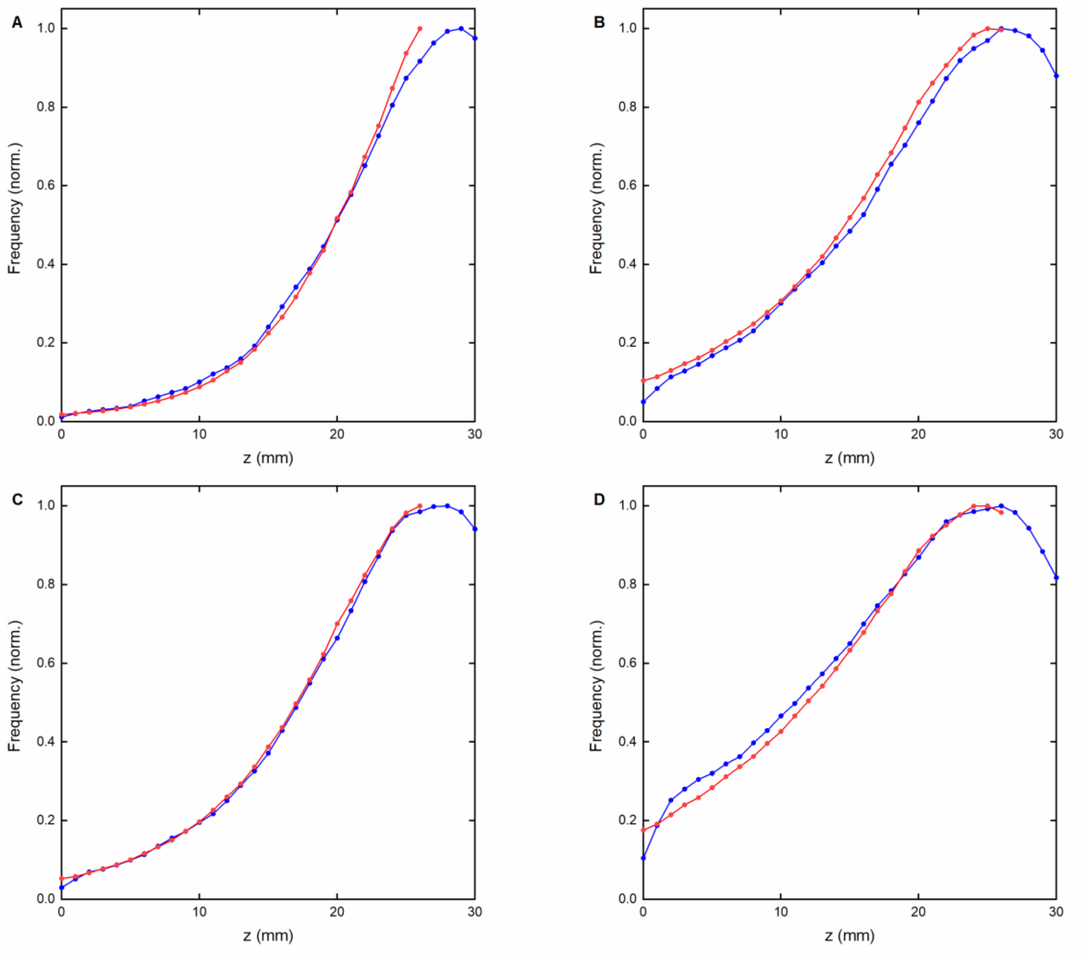


**Figure S13. Experimental (red) and theoretical (blue) distribution of monochromatic scattered light at different wavelengths and positions. (A)** 400 nm, **(B)** 500 nm, **(C)**, 600 nm, **(D)** and 700 nm. At reactor outer walls as a function of the distance to the air-suspension interface plane, as described in Figure S12.

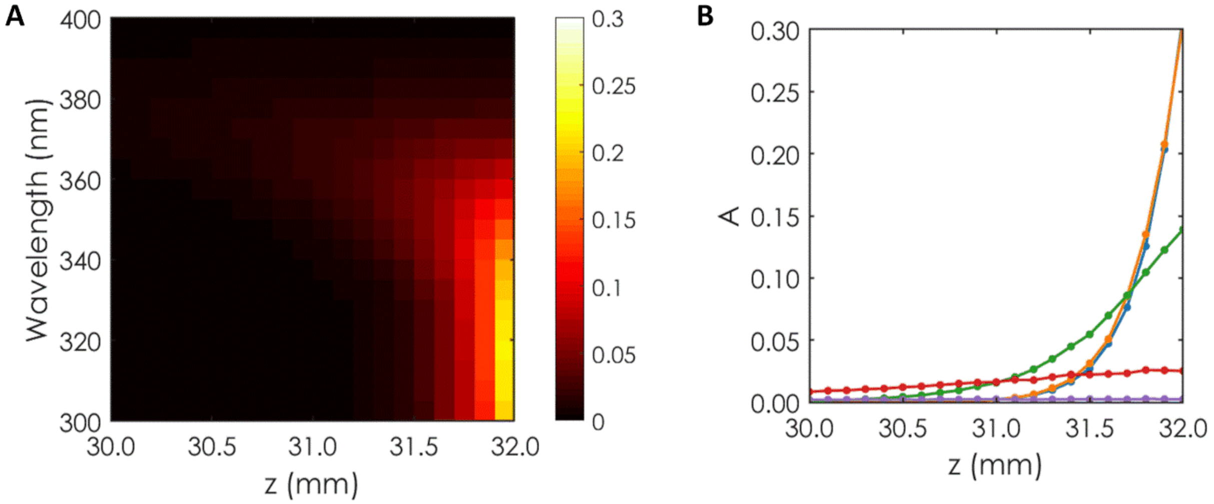


**Figure S14. Modeled solution absorptance vs depth vs wavelength. (A)** Contour plot showing the absorption profile as a function of the wavelength of the $TiO_2$ along an 80 mm reactor filled with 10 mL of the considered suspension integrating scattering centers with size distribution shown in Figure S10 and a concentration of 0.016 vol%. The air-suspension interface is located at z = 32 mm. **(B)** Absorption profile of some specific wavelengths. λ = 300 nm (blue), λ = 325 nm (orange), λ = 350 nm (green), λ = 375nm (red) and λ = 400 nm (purple).

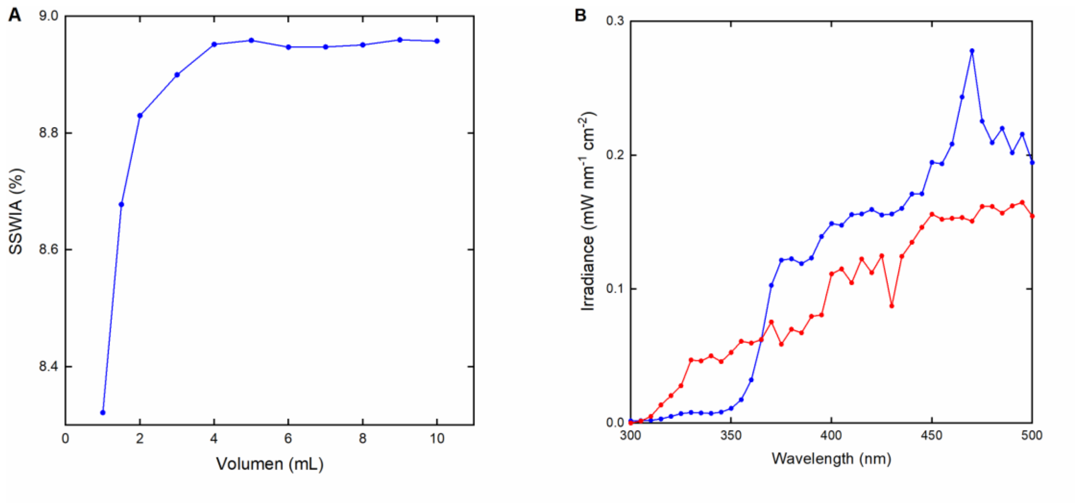


**Figure S15. Incident light and absorbed photon characterization**. **(A)** Predicted Solar Spectrum Weighted Integrated Absorptance (SSWIA) for the real system as function of the suspension volume (point data point at 10 mL had experimental validation by measuring side-scattered light as shown in Figure S12 and Figure S13). **(B)** Incident light lamp spectrum (blue) used in the study, together with AM1.5G (ASTM G-173-03) solar irradiance spectrum (red).

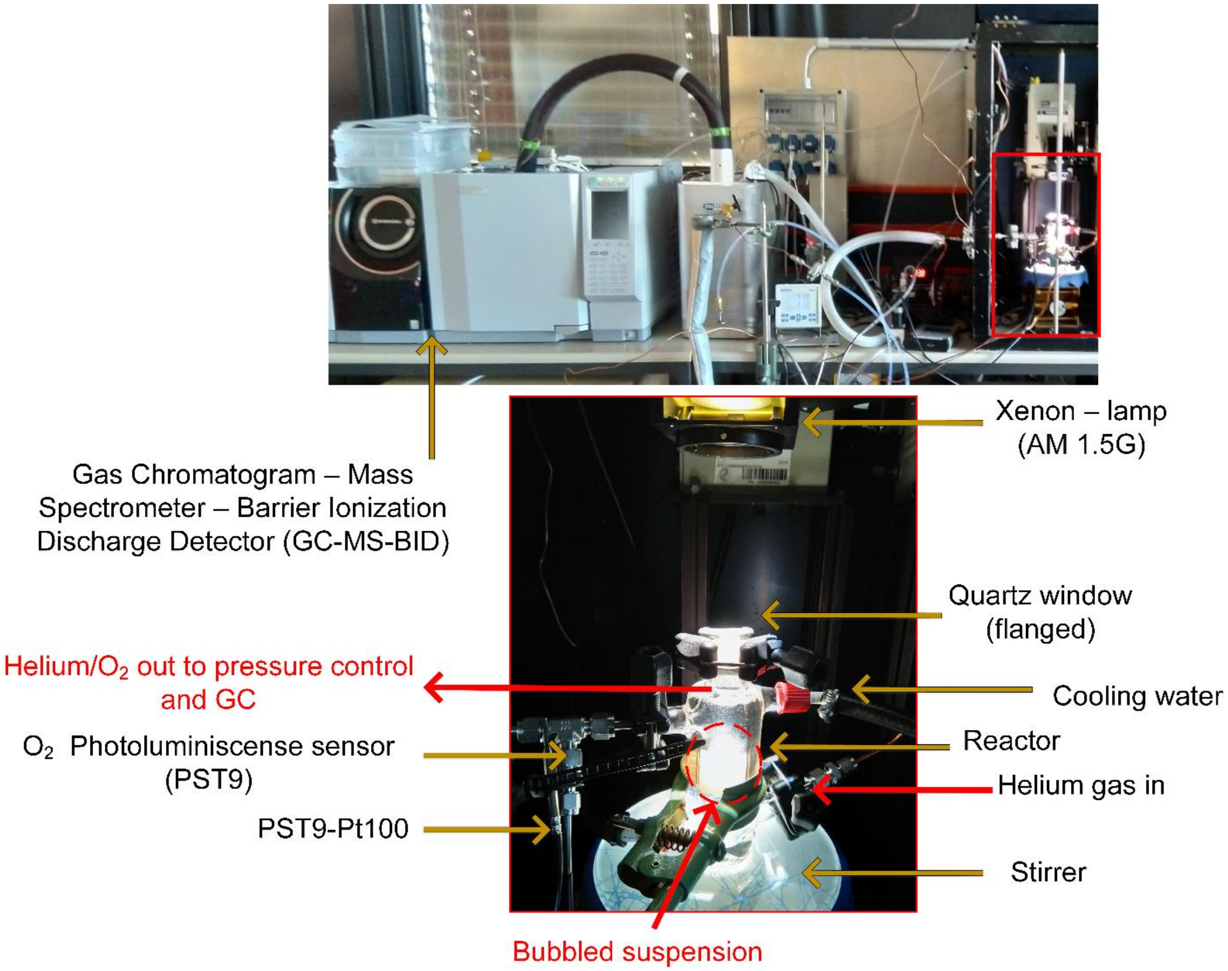


**Figure S16. Photograph of the designed photocatalytic OER continuous flow reactor assembly.**
